# Filling faults and vacancy swap occupation in half-Heusler compounds

**DOI:** 10.1093/nsr/nwag458

**Published:** 2026-07-27

**Authors:** Shen Han, Zhongkang Han, Yangjian Lin, Ting Lin, Tianqi Deng, Chenguang Fu, Xiaojuan Hu, Fu Lv, Qizhu Li, Ziheng Gao, Haonan Sheng, Yi Huang, Mengzhao Chen, Pengfei Nan, Yuhui Huang, Binghui Ge, Lin Gu, G Jeffrey Snyder, Tiejun Zhu

**Affiliations:** State Key Laboratory of Silicon and Advanced Semiconductor Materials, School of Materials Science and Engineering, Zhejiang University, Hangzhou 310058, China; State Key Laboratory of Silicon and Advanced Semiconductor Materials, School of Materials Science and Engineering, Zhejiang University, Hangzhou 310058, China; Institutes of Physical Science and Information Technology, Anhui University, Hefei 230601, China; Instrumentation and Service Center for Physical Sciences, Westlake University, Hangzhou 310030, China; Beijing National Center for Electron Microscopy and Laboratory of Advanced Materials, School of Materials Science and Engineering, Tsinghua University, Beijing 100084, China; State Key Laboratory of Silicon and Advanced Semiconductor Materials, School of Materials Science and Engineering, Zhejiang University, Hangzhou 310058, China; Institute of Advanced Semiconductors & Zhejiang Provincial Key Laboratory of Power Semiconductor Materials and Devices, Hangzhou Global Scientific and Technological Innovation Center, Zhejiang University, Hangzhou 311200, China; State Key Laboratory of Silicon and Advanced Semiconductor Materials, School of Materials Science and Engineering, Zhejiang University, Hangzhou 310058, China; State Key Laboratory of Silicon and Advanced Semiconductor Materials, School of Materials Science and Engineering, Zhejiang University, Hangzhou 310058, China; State Key Laboratory of Silicon and Advanced Semiconductor Materials, School of Materials Science and Engineering, Zhejiang University, Hangzhou 310058, China; Institutes of Physical Science and Information Technology, Anhui University, Hefei 230601, China; State Key Laboratory of Silicon and Advanced Semiconductor Materials, School of Materials Science and Engineering, Zhejiang University, Hangzhou 310058, China; State Key Laboratory of Silicon and Advanced Semiconductor Materials, School of Materials Science and Engineering, Zhejiang University, Hangzhou 310058, China; State Key Laboratory of Silicon and Advanced Semiconductor Materials, School of Materials Science and Engineering, Zhejiang University, Hangzhou 310058, China; State Key Laboratory of Silicon and Advanced Semiconductor Materials, School of Materials Science and Engineering, Zhejiang University, Hangzhou 310058, China; Institutes of Physical Science and Information Technology, Anhui University, Hefei 230601, China; State Key Laboratory of Silicon and Advanced Semiconductor Materials, School of Materials Science and Engineering, Zhejiang University, Hangzhou 310058, China; Institutes of Physical Science and Information Technology, Anhui University, Hefei 230601, China; Beijing National Center for Electron Microscopy and Laboratory of Advanced Materials, School of Materials Science and Engineering, Tsinghua University, Beijing 100084, China; Department of Materials Science and Engineering, Northwestern University, Evanston, IL 60208, USA; State Key Laboratory of Silicon and Advanced Semiconductor Materials, School of Materials Science and Engineering, Zhejiang University, Hangzhou 310058, China

**Keywords:** half-Heusler compounds, filling fault, piezoelectricity, defect–property relationship

## Abstract

Defects play a vital role in understanding and elucidating the structure–property relationship in materials science. Here, we report the existence of a new kind of planar defect, ‘filling fault’, in half-Heusler (HH) compounds—a structurally and functionally diverse family of materials. The ideal HH structure is composed of three occupied and one vacant sublattices. The two-dimensional filling fault layer tends to form with the originally vacant 4d sites occupied, fully or partly, which results in a vacancy swap occupation between the 4c and 4d sublattices. It is found that the filling faults are apt to form fully in Ni-based ZrNiSn and ScNiSb, moderately in Co-based TiCoSb and hardly in Fe-based NbFeSb and VFeSb. The unique defect configurations and the formation rule clarify the long-standing structure puzzles in HH compounds. Furthermore, we find that the filling faults and the associated vacancy swap occupation can significantly enhance the piezoelectricity and change the temperature dependence of electrical conductivity. These results deepen the understanding of precise crystallographic structures and defect–property relationships in solids, and facilitate the design of advanced materials and functional devices.

## Teaser

A newly discovered planar defect, the filling fault, in HH compounds resolves long-standing atomic-occupation puzzles and strongly enhances piezoelectricity while altering carrier transport, enabling advanced materials design.

## INTRODUCTION

Classical crystallography has laid a solid foundation in understanding and elucidating the relationship between long-range periodic atomic arrangements of crystalline solids and their macroscopic physical properties. However, real crystalline solids are usually imperfect and contain crystallographic defects such as vacancies, dislocations and boundaries. The crystallographic defects, if well controlled, could enrich the toolbox of materials scientists to exploit novel functionalities. Nevertheless, the type and configuration of the defects can vary significantly with composition and processing history, making it very challenging to identify them and fully establish their explicit relationship with macroscopic properties [1,2]. Recognition of the defects’ precise configuration sets an important step in exploiting effective defect-mediated strategies to advance their functional applications.

**Figure 1.**
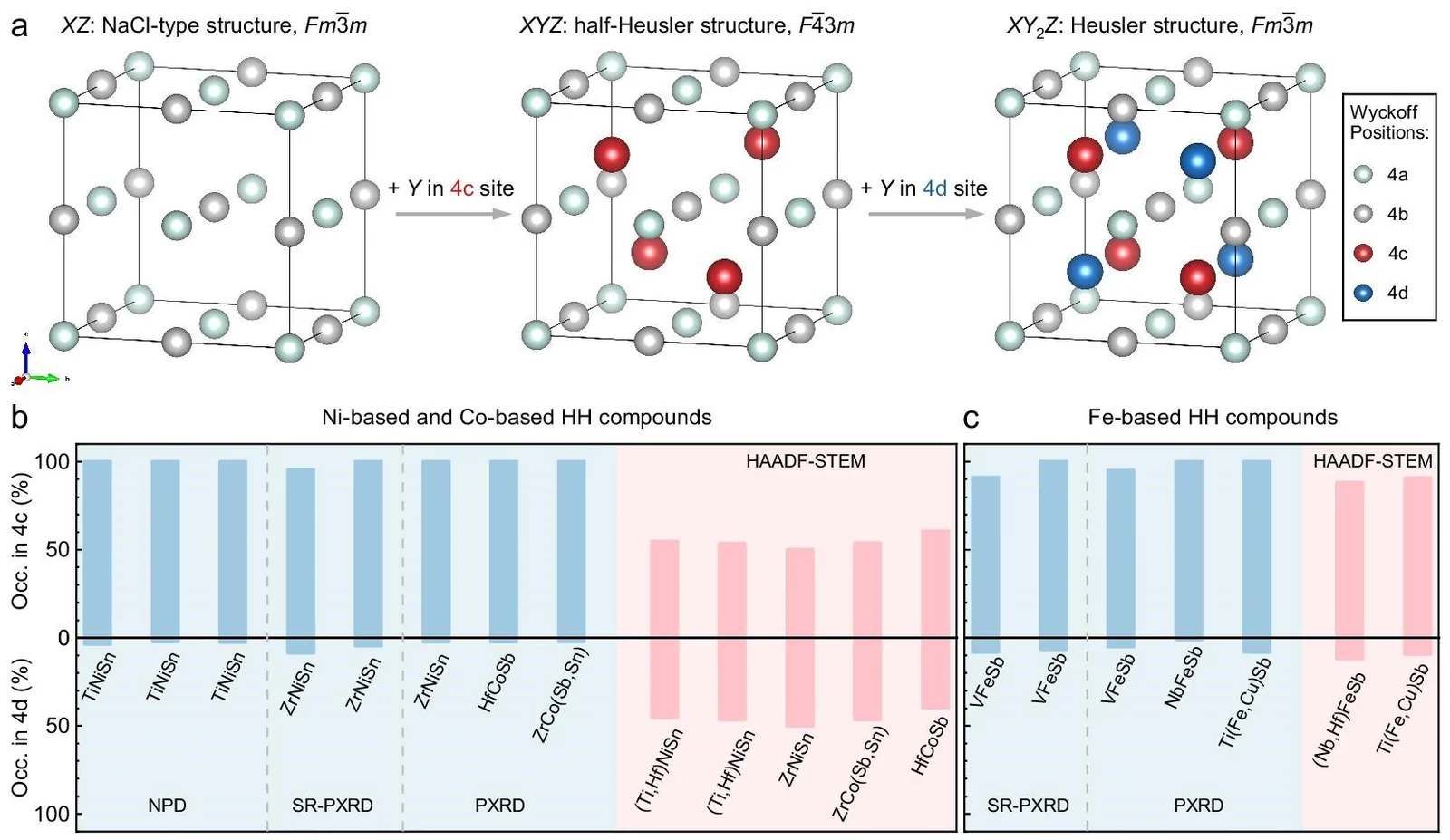
Crystal structure and atomic occupation (Occ.) of *Y* element in various HH compounds. (a) The evolution of crystal structure and Wyckoff positions for NaCl-type, HH and Heusler structures, respectively. (b and c) Summary of the literature results on the occupation of *Y* atoms in 4c and 4d sites characterized by different techniques for typical Ni- and Co-based HH compounds, as well as Fe-based HH compounds. Tables S1–S3 provide detailed data and references.

Ternary half-Heusler (HH) compounds are a big family of materials with tunable electronic structures and diverse functional properties, including but not limited to spintronics, topological insulators, superconductors and thermoelectrics [3–7], in which the inherent defects could significantly influence their physical properties [8–10]. HH compounds crystallize in a cubic structure (space group no. 216, *F*$\bar{4}$3*m*). The general formula is *XYZ*, in which *X* is the most electropositive element, *Y* is the less electropositive element and *Z* is the main group element. As schematically shown in Fig. 1a, the *X* and *Z* occupy the Wyckoff positions 4a (0, 0, 0) and 4b (1/2, 1/2, 1/2), respectively, forming a NaCl-type structure. The *Y* element occupies the Wyckoff position 4c (1/4, 1/4, 1/4). If there is an additional *Y* element filling the originally vacant 4d (3/4, 3/4, 3/4) position, the non-centrosymmetric HH structure becomes the centrosymmetric Heusler structure with a general formula of *XY*_2_*Z* [11].

The vacant 4d site in the HH structure provides a huge tunability for accommodating additional homogeneous or heterogeneous elements utilized to regulate the physical properties [4,12]. However, the actual occupation of the *Y* element at 4c/4d sites in HH compounds has long been puzzling over the past two decades, giving rise to two contradictory viewpoints. On one hand, the diffraction spectroscopy methods, which provide a general description of atomic occupation in HH compounds [13,14], present the result that the excess *Y* atoms occupy the 4d sites in a random disorder way. On the other hand, the high-angle annular-dark-field scanning transmission electron microscopy (HAADF-STEM) observation, directly presenting the local atomic distribution, suggests that *Y* atoms could partly occupy both 4c and 4d sites by forming a Heusler-like structure [15–18], i.e. in a correlated disorder way. The distinct atomic arrangement of random and correlated disorders in crystalline solids leads to their remarkably different physical properties [1,19,20]. Thus, clarifying the precise configuration of crystallographic defects in HH compounds is of great importance for designing the functionalities of this big material family.

A comparative analysis of the Fe-, Co- and Ni-based HH compounds, i.e. *X*Fe*Z, X*Co*Z* and *X*Ni*Z*, helps understand the two different viewpoints on the atomic occupation of the *Y* element. Various chemical composition analysis techniques, including electron probe microanalysis (EPMA), energy-dispersive X-ray spectroscopy (EDS) and inductively coupled plasma spectroscopy, suggest that there should be excess Co and Ni of ∼1%–6% in *X*Co*Z* and *X*Ni*Z* (Table S1), and the actual compositions become *XY*_1+_*_δ_Z* (*Y* = Co, Ni, 1% ≤ *δ* ≤ 6%). According to the structural refinements based on the synchrotron radiation powder X-ray and neutron powder diffractions, the 4c site is nearly fully occupied by *Y* (nearly 100%) with the excess *Y* (∼1%–9%) occupying the originally vacant 4d site (Table S2), pointing to a random disorder way. In contrast, the HAADF-STEM results suggest a nearly equal occupation of *Y* in both the 4c and 4d sites (almost 50% vs 50%) in the *X*Co_1+_*_δ_Z* and *X*Ni_1+_*_δ_Z* (Table S3), pointing to a correlated disorder way, as summarized in Fig. 1b. For the Fe-based *X*Fe_1+_*_δ_Z* HH compounds, the composition, spectroscopy and microscopy analysis (Tables S1–S3) on the *Y* occupation give a consistent conclusion. Namely, the 4c sites are nearly fully occupied by Fe atoms (nearly 100%), while a slight amount of excess Fe (∼1%–8%) occupies the 4d sites (Fig. 1c). These results indicate that the occupation of *Y* atoms in HH compounds varies with specific *Y* constituents, and the precise occupation of *Y* atoms in HH compounds is still elusive.

In this article, we find the existence of a new kind of planar defects in the Ni- and Co-based HH compounds, which is named as ‘filling faults’ by analogy with the stacking faults in crystals. The two-dimensional (2D) filling fault layers with the *Y* element occupying both the 4c and 4d sites tend to form at the boundaries of the region with the *Y* element at 4c sites, and a symmetrical region with the swap occupation of *Y* element at originally vacant 4d sites will correspondingly occur. The finding of filling faults and the swap occupation clarifies the contradictory observation on the atomic occupations in HH compounds by diffraction spectroscopy and HAADF-STEM methods. Furthermore, we find that the filling faults and swap occupation can significantly enhance the piezoelectric response and change the temperature dependence of electrical conductivity in the HH compounds. These results deepen the understanding of defect–property relationships in solids, facilitating the design of advanced materials and functional devices.

## RESULTS

### HAADF-STEM observations

**Figure 2.**
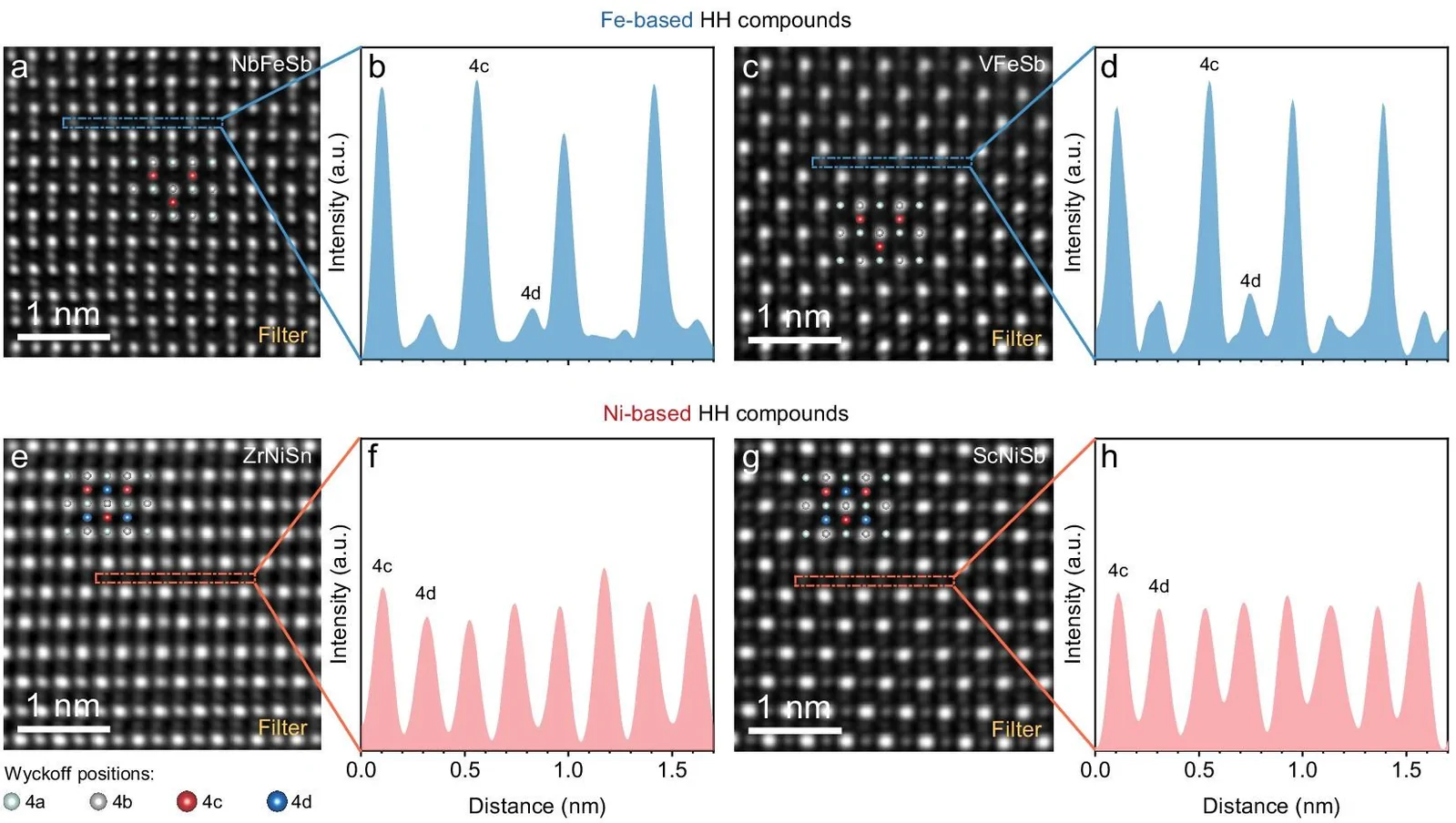
Occupation of transition metal *Y* in Ni-based and Fe-based HH compounds. (a, c, e and g) HAADF-STEM images along the [110] direction for NbFeSb, VFeSb, ZrNiSn and ScNiSb, respectively. Ball markers with different colors are used to distinguish different Wyckoff positions. (b, d, f and h) Intensity profiles corresponding to areas in (a, c, e and g) indicated by the dashed lines.

To study the precise atomic occupations in HH compounds, we performed direct HAADF-STEM observation on a series of compositions, including the Ni-based ZrNiSn and ScNiSb, Co-based TiCoSb and Fe-based VFeSb and NbFeSb. The phase purity and chemical composition uniformity of all the samples were examined by X-ray diffraction (XRD), EPMA and EDS mapping (Tables S4 and S5, Figs S1 and S2), showing that the HH phase was obtained without obvious secondary phases. The slight excess of transition metal *Y* in ZrNiSn, TiCoSb, NbFeSb and VFeSb was detected, while a deficiency of *Y* occurred in ScNiSb. The atomic-resolution images of NbFeSb and VFeSb are shown in Fig. 2a and c with the low-magnification transmission electron microscopy (TEM) images and selected area electron diffraction (SAED) patterns along [110] direction presented in Fig. S3. It is observed that the Fe atoms predominantly occupy the 4c site, pointing to a nearly ideal HH crystal structure. The intensity profile (Fig. 2b and d) shows that most Fe atoms occupy the 4c site with very few excess Fe (Table S5) occupying the 4d site.

A remarkably distinct result was observed for ZrNiSn and ScNiSb. The HAADF-STEM image (Fig. 2e) and the intensity profile (Fig. 2f) of ZrNiSn, with excess Ni of ∼6% in its actual composition, suggests a nearly equal occupation of Ni at 4c and 4d sites, consistent with previous reports [15,18,21] (Fig. 1b). As for ScNiSb with an actual composition of Ni deficiency (Table S5), a nearly equal Ni occupation in 4c and 4d sites was also observed (Fig. 2g and h), indicating that this may be a common phenomenon in Ni-based HH compounds. It is worth emphasizing that the above results were not obtained in specially selected regions but uniformly in the samples, as demonstrated in larger regions presented in Fig. S4. The remarkably distinct 4c/4d occupations between Ni-based and Fe-based HH compounds highlight its possible correlation with the species of the transition metal *Y* in HH compounds.

### Monte Carlo simulations

**Figure 3.**
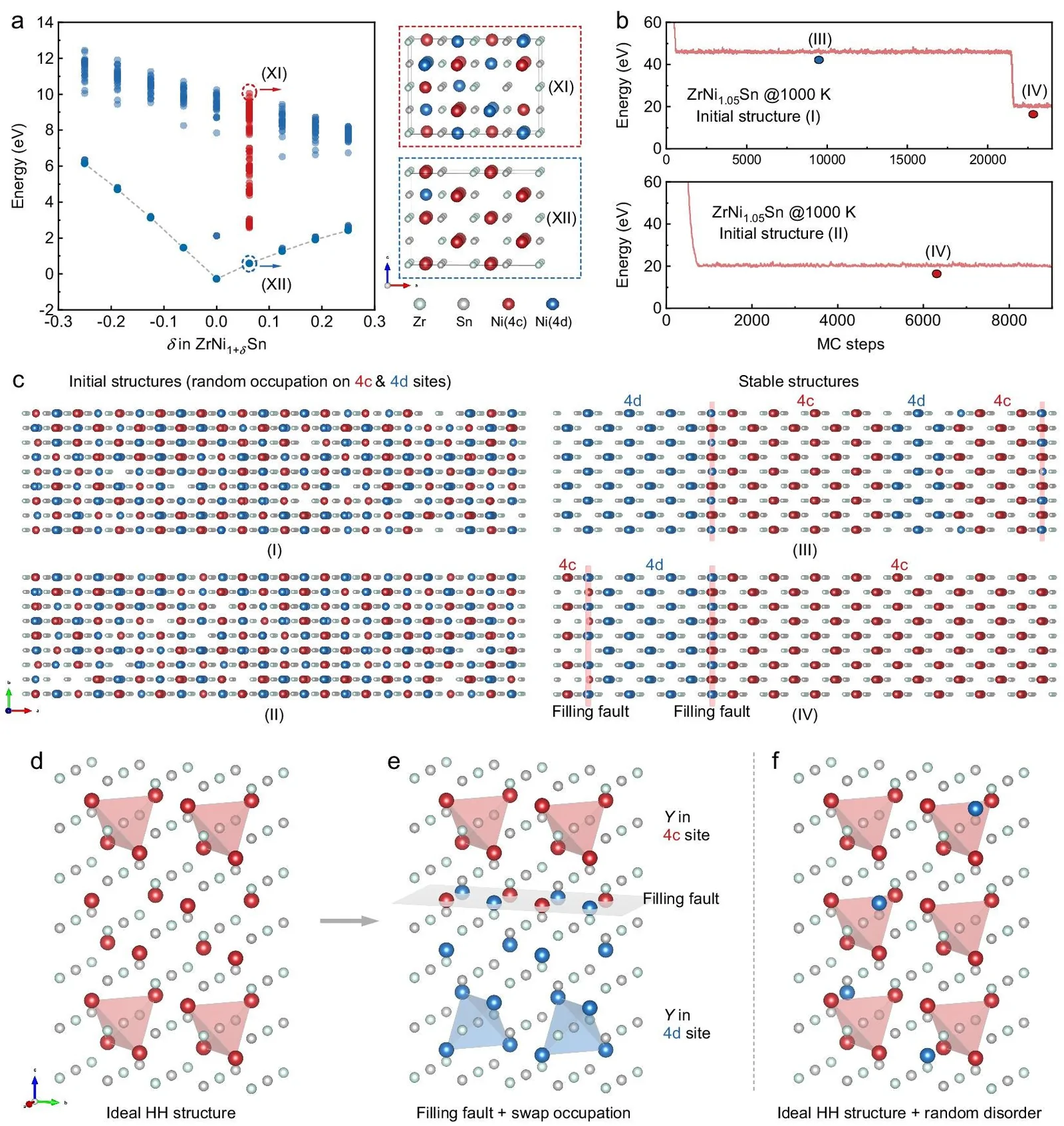
Calculations on the atomic occupation of Ni in ZrNi_1+_*_δ_*Sn. (a) Calculated formation energy for ZrNi_1+_*_δ_*Sn with different Ni concentrations. Atomic occupations are given for structures with the highest energy (XI) and lowest energy (XII) in the ZrNi_1.0625_Sn composition. (b) Formation energy changes of MC simulation for Ni atoms' occupancy in ZrNi_1.05_Sn at 1000 K. (c) Atom occupations for initial structures (I) & (II), stage-stable structure (III) and terminal-stable structure (IV) in panel (b). To facilitate the visualization of 4c and 4d sites, the directions for all structures in panels (a–c) were realigned, with ab and ac planes transformed from the initial (100) crystallographic plane family to the (110) family. (d–f) Schematic diagram of the swap occupation of *Y* element induced by filling fault (d and e) and randomly disordered *Y* element at 4d sites (f).

We further carried out theoretical calculations on ZrNi_1+_*_δ_*Sn (−0.25 ≤ *δ* ≤ 0.25) to visually understand the occupation of *Y* atoms in HH compounds. The formation energies of various ZrNi_1+_*_δ_*Sn structures with different Ni concentrations were first obtained by the first-principles calculations (Fig. 3a), by fixing Zr and Sn atoms at their lattice positions and letting Ni atoms randomly locate at either 4c or 4d site. Compared to that of the ideal HH structure with *δ* = 0, the calculated formation energy of all the studied hundreds of structures increases no matter whether *δ* > 0 (Ni excess) or *δ* < 0 (Ni deficiency). A subset of structures exhibits markedly lower formation energies across all *δ* values (connected by the dashed line). Representatively, the Ni occupation for the structures with the highest energy (XI) and the lowest energy (XII) in ZrNi_1.0625_Sn (a composition close to the actual composition in experiments) is shown in Fig. 3a. Compared to the highest energy structure (XI), where Ni randomly occupies both 4c and 4d sites, the distinct feature of the lowest energy structure (XII) is that Ni tends to preferentially occupy the 4c site with the excess Ni occupying the 4d site. This result supports the structural refinement results from diffraction spectroscopy analysis (Fig. 1b) but again leads to the HAADF-STEM observation being very puzzling, as the latter suggests a random occupation of Ni at both 4c and 4d sites and points to the unstable highest energy structure (XI).

The processing history can significantly affect the atomic occupation in real crystals. Hence, we further carried out Monte Carlo (MC) simulations by including the temperature effect. The structures shown in Fig. 3a are used to train the prediction of formation energy by a compressed sensing-assisted Cluster Expansion (CE) approach [22]. The CE approach helps to enable the construction of scales large enough to reflect the precise atomic occupation while reducing the burden on computational resources. Cross-validation of predicted energies (Fig. S5) confirms the favorable prediction accuracy of the model.

As shown in Fig. 3b, the energy change started from two different initial structures during MC simulations of ZrNi_1.05_Sn at 1000 K. Four million steps were simulated, and only effective steps were extracted and counted. A total of 404 Ni atoms are allowed to freely occupy 384 4c sites and 384 4d sites, at a sufficiently large scale to reflect real occupancy. For the initial structure (I) with a complete random occupation of Ni in either 4c or 4d sites (Fig. 3c), the formation energy first decreases to reach a stage-stable structure (III), as shown in Fig. 3b. Ni atoms in stage-stable structure (III) have been shown to preferentially occupy one set of sublattice (either 4c or 4d) in a certain region, respectively. Moreover, between the 4c and 4d regions, it is found that a few excess Ni atoms fill both 4c and 4d sites by forming a special 2D single layer, as shaded in light red in Fig. 3c. With further training, a terminal-stable structure (IV) is obtained, in which Ni atoms only occupy 4c or 4d sublattices in a certain region and form a 2D single layer in between the 4c and 4d regions.

A different initial structure (II) with a complete random occupation of Ni in either 4c or 4d sites (Fig. 3c) was also used for carrying out the simulation, and it finally evolved to the same terminal-stable structure (IV) as that of the initial structure (I). The convergence of different initial structures (I and II) to the same terminal state (IV) implies that the structure (IV) can reflect the actual occupation of Ni atoms in ZrNi_1.05_Sn at 1000 K. Given that experimental samples predominantly remain at room temperature during characterization, we initiated an additional simulation with ten million steps to implement a temperature ramp from 1000 to 300 K. The terminal state (IV) was chosen as the new initial structure, and the energy showed no significant variation with increasing effective steps (Fig. S6), demonstrating that the occupation of Ni atoms shown in Fig. 3c remains representative across a broad temperature range.

Accordingly, we can generalize two key features of the unique *Y* occupation in Ni-based HH compounds: (i) Ni atoms tend to occupy either 4c or 4d sublattice in a certain region and (ii) in between the separate 4c and 4d regions, there exists a unique 2D layer in which both 4c and 4d sites are occupied. To the best of our knowledge, such a 2D layer defect with filled atoms on the originally vacant sites has never been reported in previous studies and textbooks. Hence, it is a new kind of planar defect, named as ‘filling fault’ hereafter, by analogy with the classical planar stacking fault in crystalline solids. It is worth noting that comparison of the concepts between these two types of planar defect helps to further clarify the distinctive features of filling faults. A stacking fault refers to a defect formed by a local disruption in the layering sequence when atomic layers are ranged in a periodic order and represents a local deviation from the ideal stacking order. In contrast, the planar filling fault involves the localized filling of originally vacant sublattice with the constituent atoms. The key characteristic of filling fault is its association with originally vacant sublattices that can form a low energy stable state when filled by the constituent atoms. If other material systems contain vacant sublattices with features similar to the 4d sublattice in the HH structure, filling faults may also be observed in them.

Now, we can summarize the occupation characteristics of *Y* atoms in HH compounds with the filling faults. The discussion starts with an ideal HH structure (Fig. 3d). The filling fault is formed with the slight excess *Y* atoms occupying the originally vacant 4d site, and the presence of a filling fault could induce a new type of atomic occupation: the filling fault acts as a boundary of the region with *Y* atoms occupying 4c sites, and a symmetrical region with the swap occupation of *Y* atoms at 4d sites occurs, as schematically shown in Fig. 3e. In this case, the filling fault acts as a low-energy transition interface ensuring the coordination stability of the *X* and *Z* atoms even when the *Y* atoms on both sides occupy two different sets of sublattices.

Figure 3f illustrates the long-standing conventional view: the slight excess *Y* atoms occupy partial 4d sites in random disorder while maintaining the ideal HH structure. This stands in stark contrast to the occupation patterns we reveal in Fig. 3e. Actually, such filling-fault-induced unique swap occupation of *Y* atoms well explains the previous controversial results obtained from the diffraction spectroscopy and the electron microscopy methods, as summarized in Fig. 1b. Regardless of any side of the vacancy swap occupation, *Y* atoms occupy only one complete set of sublattices, consistent with the diffraction spectroscopy result that *Y* atoms occupy mainly at the 4c sites (owing to the crystallographic equivalence of 4c and 4d sites in the HH lattice). In contrast, for HAADF-STEM, the original 4c occupation and the filling-fault-induced 4d occupation are specifically identified, giving a nearly equal occupation of *Y* atoms at 4c and 4d sites.

### Direct observation of filling fault

**Figure 4.**
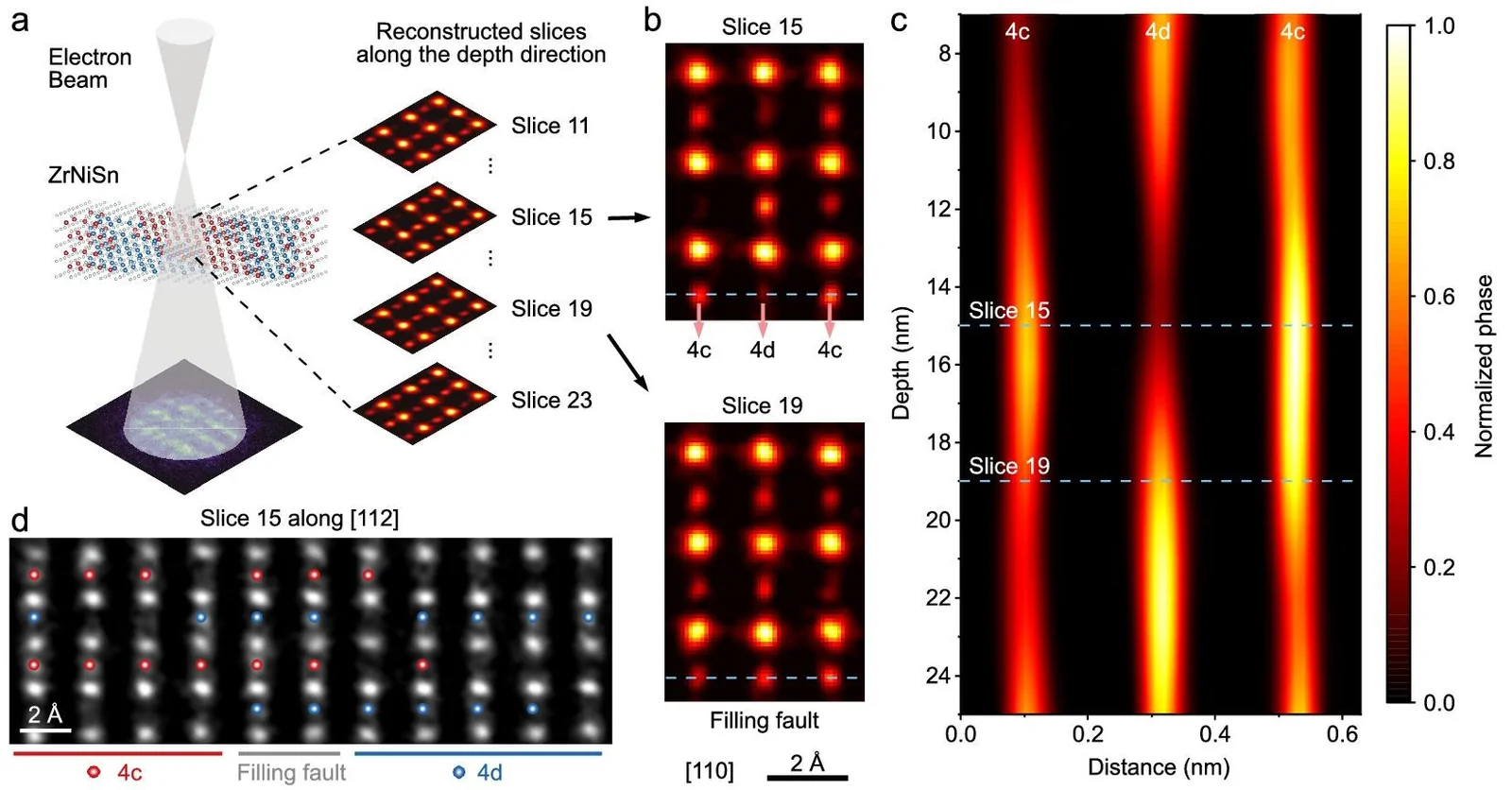
Direct observation of filling faults in ZrNiSn by 4D-STEM. (a) Schematic of the defocused scanning and three-dimensional reconstruction with MEP. (b) Phase image of slices along the [110] direction. (c) Depth profile along the dashed lines in panel (b). (d) Phase image of a slice along the [112] direction.

The MC simulations for the atomic occupation of excess Ni in ZrNiSn (Fig. 3c) reveal the novel planar defect filling fault and the associated vacancy swap occupation, which represent an energetically stable final state, whereas real samples undergo complex synthesis processes and may not fully reflect such idealized conditions. To gain a more direct recognition of the filling faults, we further carried out additional characterization using the recently established energy-filtered multislice electron ptychography (MEP) technique based on advanced four-dimensional STEM (4D-STEM). Such technique reconstructs a three-dimensional object with depth information by scanning sample with a defocused electron probe (Fig. 4a, details in ‘Methods’ section), enabling the acquisition of nanometer-thick slices suitable for detailed observation of atomic-scale defects [23,24].

We performed effective MEP slice reconstruction on ZrNiSn with a depth of 1 nm for each slice. The reconstruction was first performed along the [110] direction, corresponding to the orientation used in the above HAADF-STEM observations (Fig. 2). Figure 4b shows phase maps of the same region along [110] direction at different slice numbers, where the occupation of Zr and Sn atoms remains unchanged. In the image at slice 15, Ni atoms preferentially occupy the 4c sites, exhibiting a nearly ideal HH structure within the thin layer. In contrast, at slice 19, Ni atoms are uniformly distributed between the 4c and 4d sites, indicating the formation of a filling fault. The complete reconstructed slices for this region are presented in Fig. S7, showing the continuous variation in Ni atoms and the gradual transition from the ideal HH structure to the filling fault.

The depth resolution of the slice reconstruction provides further corroborative evidence. Figure 4c displays the phase profile along the depth direction, taken along the position marked by the blue dashed lines in Fig. 4b. Around slice 15, a strong phase signal is observed primarily at the 4c sites, while the signal at the 4d sites is weak. As the slice number increases, the phase of 4c sites decreases while the 4d sites increase, where the transition layers correspond to a filling fault, at around slice 19. This transition between the ideal HH structure and filling fault along the depth direction could not be an isolated case, with reconstructed slices and depth profiles for additional region shown in Fig. S8.

To gain a more comprehensive display of this novel defect, Fig. 4d displays phase image extracted along [112] direction, where the 4c and 4d sites can be directly distinguished as they are separated at alternative planes (see schematic in Fig. S9). Within a slice containing only 3–4 atomic layers, we directly observed the transition of Ni atoms from occupying the 4c sites to 4d sites. In the transition region, as predicted by the simulations, there exists a layer with co-occupation in 4c and 4d sites, corresponding to the filling fault. The filling fault and related vacancy swap occupation on both sides are observed across different slices (Fig. S10). Partially deviated from the final stable state in MC simulations (Fig. 3c-IV), the 4c and 4d occupations and the filling fault in the real sample are not perfectly clean. This makes sense considering that the real synthesis conditions are non-equilibrium and the characterizations were performed at room temperature. These experimental observations of filling faults and surrounding 4c and 4d occupations provide direct evidence for the novel planar defect and the vacancy swap occupation.

### Incomplete filling fault

**Figure 5.**
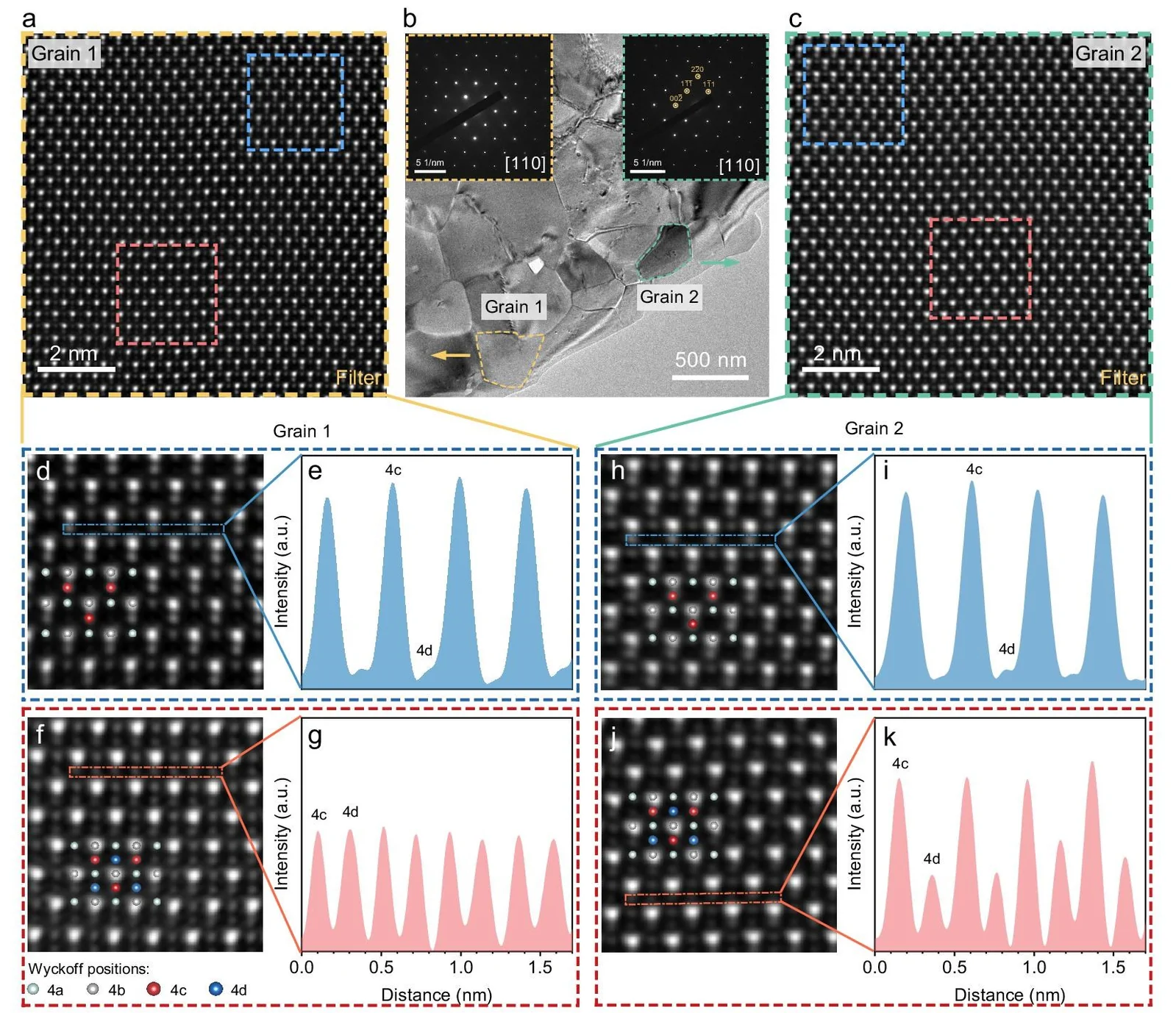
Occupation of Co atoms in TiCoSb. (a, c) HAADF-STEM images of TiCoSb in different grains indicated by yellow (Grain 1) and green (Grain 2) dashed lines in panel (b), respectively. (b) Low-magnification TEM images of TiCoSb with the corresponding [110] direction SAED pattern as inset. (d, f, h and j) Enlarged HAADF-STEM images corresponding to areas indicated by red and blue dashed lines in panels (a) and (c), respectively. Ball markers with different colors are used to distinguish different Wyckoff positions. (e, g, i and k) Intensity profiles corresponding to areas in (d, f, h and j) indicated by dashed lines, respectively.

The filling faults and the vacancy swap occupation in Ni-based HH compounds disappear when the *Y* changes from Ni to Fe (Fig. 2). Co locates between Fe and Ni in the periodic table of elements, and its distribution in *X*Co*Z* HH compounds deserves further investigation, which is beneficial for understanding the formation rule of filling faults. The STEM image of TiCoSb is shown in Fig. 5. Two grains were observed (labeled as Grain 1 and 2, in Fig. 5b) with the SAED patterns in the inset of Fig. 5b and HAADF-STEM images in Fig. 5a and c. Distinct from the Ni- and Fe-based HH compounds, the occupation of Co atoms shows different configurations even within the same grain. For Grain 1 (Fig. 5a), the image contrast at 4d site was not observed in the region denoted by the blue dashed box (the magnified image in Fig. 5d). In contrast, the Co atoms occupy both 4c and 4d sites in another region highlighted by the red dashed box (the magnified image in Fig. 5f). The atomic intensity profiles (Fig. 5e and g) further confirm the difference in the occupation of Co atoms in two regions within the same grain, i.e. one region exhibits a nearly ideal HH structure while the other presents a filling-fault-induced vacancy swap occupation.

Owing to the complexity of Co occupations in TiCoSb, another grain (Grain 2 in Fig. 5b) was chosen for double check. Different occupations of Co atoms were also observed (magnified images in Fig. 5h and j). Atomic intensity profiles in Fig. 5i and k demonstrate qualitatively similar but quantitatively different results from Grain 1. The blue region in Grain 2 still exhibits the ideal HH structure, and the red region exhibits simultaneous occupation at the 4c and 4d sites. It is noteworthy that the atomic intensity of the 4d site is remarkably lower than that of the 4c site, different from the observation for Grain 1 (Fig. 5g).

The filling faults in the HH structure can be viewed as the Heusler layer. Thus, analyzing the formation enthalpy of the Heusler structure could provide the insight into the formation mechanism of the filling faults in HH compounds with different *Y* elements. Table S6 shows the calculated formation enthalpy for ZrNiSn, TiCoSb and NbFeSb, as well as their corresponding Heusler compositions ZrNi_2_Sn, TiCo_2_Sb and NbFe_2_Sb. The ZrNiSn, TiCoSb and NbFeSb have the negative formation enthalpy, indicating their stability. For their corresponding Heusler compositions, the formation enthalpy of ZrNi_2_Sn (−0.527 eV/atom) is the lowest, indicating that the ZrNi_2_Sn is more stable and can be synthesized (Supplementary Note S1 and Fig. S11). This explains why the filling faults can stably exist in ZrNiSn and act as a transition interface for the vacancy swap occupation (Fig. 2e and f). Differently, NbFe_2_Sb has a nearly zero formation enthalpy (−0.029 eV/atom), indicating the instability of this composition (Supplementary Note S1), which explains why NbFeSb maintains an ideal HH structure without vacancy swap occupation (Fig. 2a and b).

The formation enthalpy of TiCo_2_Sb is between those of stable ZrNi_2_Sn and unstable NbFe_2_Sb, but its pure phase was also not obtained in the experiment. Even so, the compositional characterization suggests a matrix composition of TiCo_1.2–1.4_Sb (Supplementary Note S1). Compared with the formation enthalpy of TiCo_2_Sb (−0.272 eV/atom), TiCo_1.5_Sb has an even lower value of −0.403 eV/atom, implying the incomplete filling faults with partly occupied 4d sites in TiCoSb. Such incomplete filling faults may lead to a reduction in the areal fraction of vacancy swap occupation, which explains the varied 4c/4d intensities observed in Fig. 5. The incomplete filling fault in Co-based HH compounds deepens the understanding of this new kind of planar defects. In short, the sequential change of *Y* from Ni to Co to Fe drives a transition of filling faults in HH compounds: the complete filling fault with both 4c and 4d sites occupied in Ni-based HH compounds, incompletely filled 4d sites in Co-based compounds and no filling faults in Fe-based HH compounds. This trend establishes the *Y*-element-dependent formation rule of filling faults in HH compounds.

### Filling-fault-associated physical properties

**Figure 6.**
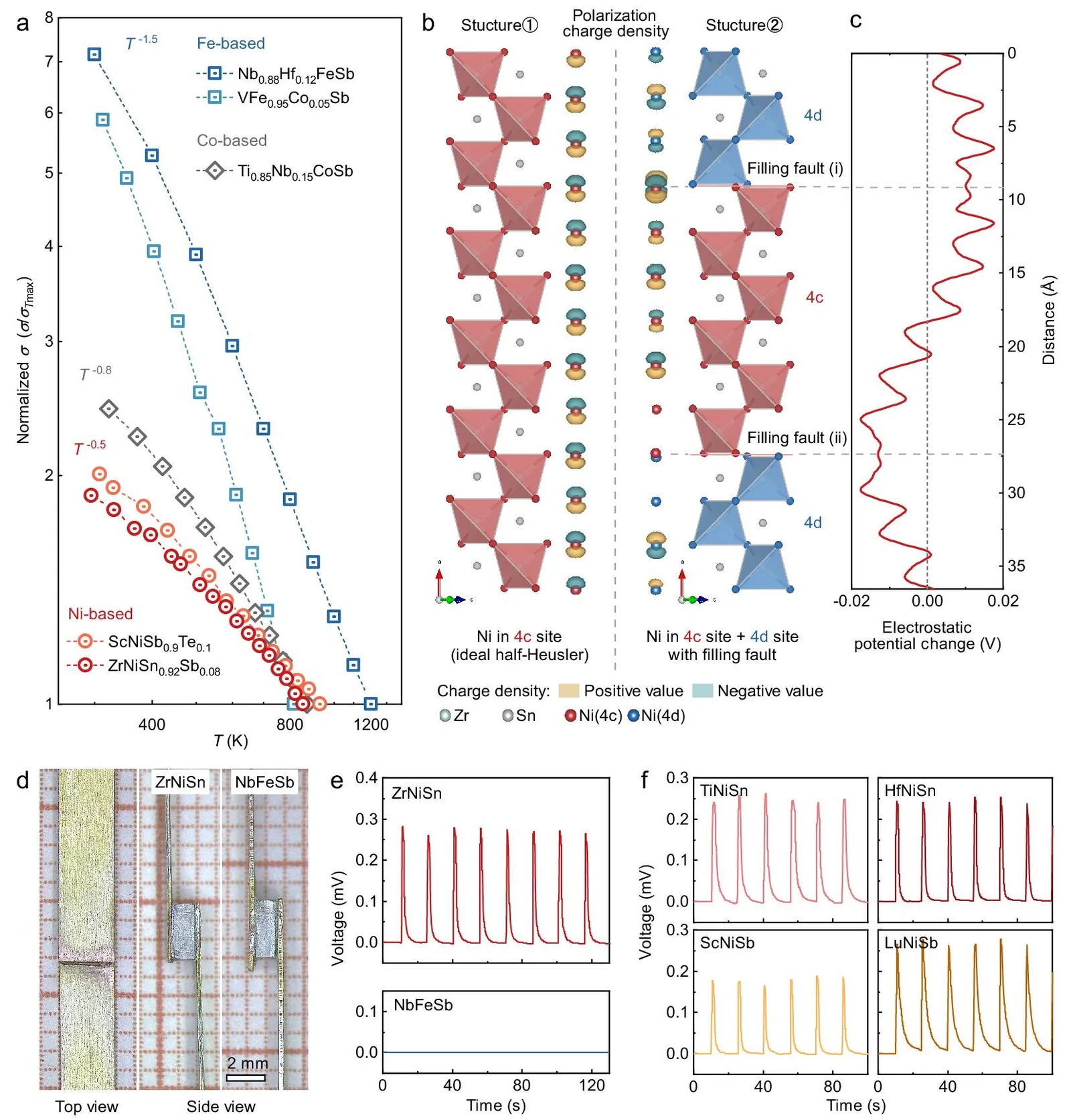
Effects of filling fault on physical properties in HH compounds. (a) Temperature-dependent electrical conductivity for Ni, Co and Fe-based HH compounds [25–29]. (b) Calculation for strain-induced polarization in ZrNiSn with ideal HH structure (structure①) and swap occupation with filling faults (structure②). The closed surface in the structure diagram refers to the tetrahedral coordination of Zr atoms with the nearest neighboring Ni atoms in 4c (red) or 4d (blue) sites. The yellow and green shades correspond to positive and negative charge density at Ni atoms in 0.002 e/bohr isosurface values, respectively. (c) Calculated electrostatic potential change with the atomic position for structure② in panel (b). (d) Optical images for piezoelectric sensors made by polycrystalline ZrNiSn and NbFeSb. (e and f) Time-dependent open-circuit voltage for piezoelectric sensors made by polycrystalline ZrNiSn, NbFeSb and four other Ni-based HH compounds, respectively, under finger-pressing pressure.

The filling fault and its progressive evolution with *Y* elements for HH compounds provide a view to understand their different physical properties. The temperature dependence of electrical conductivity, which manifests the carrier scattering mechanism, was found remarkably different for Ni-, Co- and Fe-based HH compounds [25–29]. As shown in Fig. 6a, Ni-based HH compounds ZrNiSn and ScNiSb exhibit much weaker temperature-dependent electrical conductivity (*σ* ∼ *T*  ^−0.5^), compared to Fe-based HH compounds NbFeSb and VFeSb (*σ* ∼ *T*  ^−1.5^). The observed *σ* ∼ *T*  ^−1.5^ suggests that the carrier transport is dominated by acoustic phonon scattering [30]. Nevertheless, the weaker temperature-dependent *σ* in Ni- and Co-based HH compounds implies the additional carrier-scattering sources besides the phonons, which should be associated with the observed filling faults.

Since it is very challenging to prepare HH compounds with the same composition but different defect states (with and without filling faults), to strengthen the understanding of the experimentally observed filling-faults-weakened carrier transport, we further carried out the first-principles calculations to comprehensively evaluate the effects of different scattering mechanisms on carrier transport in ZrNiSn with and without filling faults. We considered two possible cases, i.e. random disorder and filling faults, when including additional scattering resources relative to the 5% excess Ni in ZrNiSn. To make a more comprehensive consideration, two compositions with different carrier concentrations were selected for performing the calculations. The results were derived directly from first-principles calculations and involved no parameter fitting. As presented in Fig. S12 (more detailed discussions provided in Supplementary Note S2), the experimental data fail to align with the calculated total mobility when random disorder is included but match well with the calculated one when filling faults are considered. These results strengthen the filling-fault-mediated electrical transport properties in Ni-based HH compounds.

In addition to the impact on carrier transport, the filling fault and the vacancy swap occupation may induce polarization and influence the piezoelectric properties. The piezoelectricity in HH compounds has been theoretically predicted [31], and recently experimentally observed in the single crystalline HH crystals TiNiSn, ZrNiSn and TiCoSb [32]. One puzzling phenomenon is that the experimentally observed piezoelectric strain coefficient in ZrNiSn single crystal (*d*_14_ ∼ 38 pC/N) is much larger than the theoretically predicted one (*d*_14_ ∼ 1.5 pC/N) [32]. Since piezoelectric properties can be enhanced by crystallographic defects [33,34], the relationship between the piezoelectric response and the currently identified filling faults in HH compounds deserves further study.

We constructed two different ZrNiSn structures and calculated the corresponding strain-induced polarization. One structure is the ideal HH structure with Ni at the 4c sites (structure① in Fig. 6b), and the other is a sandwich-like structure for Ni occupation along an order ‘4d–filling fault (i)–4c–filling fault (ii)–4d’ (structure② in Fig. 6b). The application of strain within the bc-plane induces polarization along the a-axis in these two structures. Given the centrosymmetric nature of the Zr- and Sn-occupied sublattices, which preclude net polarization contributions (Fig. S13), only the polarization charge density localized around Ni atoms is visualized in Fig. 6b. Compared with the ideal HH structure, the filling fault results in the change of the strain-induced polarization charge (Fig. 6b), in which charge accumulates on filling fault (i) and depletes on filling fault (ii). Figure 6c shows the calculated electrostatic potential corresponding to the structure in Fig. 6b. Starting from the top 4d region, the electrostatic potential overall rises when approaching the filling fault (i). Since the 4c and 4d sublattices contribute opposite polarization directions, the electrostatic potential then decreases in the 4c region and reaches a minimal value near the filling fault (ii). The disparity in electrostatic potential drives the redistribution of polarization charges from filling fault (ii) to filling fault (i), and the resulting charge density contrast engenders emergent polarization that spans beyond atomic and unit cell scales.

We measured the piezoelectric coefficient *d*_eff_ in polycrystalline ZrNiSn samples with filling faults, which is ∼10–13 pC/N at room temperature. The results for samples with different sizes verify that the measured *d*_eff_ values are repeatable (Fig. S14). Noteworthily, for piezoelectric materials without spontaneous polarization, the piezoelectric response generally vanishes when the materials are made in their polycrystalline formality, due to the random orientation of the grains [35]. It is thus surprising that a decent piezoelectric coefficient can be detectable in polycrystalline ZrNiSn. Further, we made piezoelectric sensors using polycrystalline ZrNiSn (with filling faults) and NbFeSb (without filling faults), respectively (Fig. 6d). As shown in Fig. 6e, the ZrNiSn-based piezoelectric sensor presents a stable piezoelectric response, while no obvious piezoelectric response was detected for the NbFeSb-based sensor.

Furthermore, to strengthen the generality of the observed defect-mediated piezoelectricity, we supplemented piezoelectric experiments on four additional Ni-based HH compounds, i.e. TiNiSn, HfNiSn, ScNiSb and LuNiSb. As shown in Fig. 6f and Fig. S15, obvious piezoelectric response can be observed in all these Ni-based HH compounds. These results suggest that the piezoelectric response in polycrystalline HH compounds is associated with the filling faults and vacancy swap occupation. Combined with the polarization domains induced by filling faults (calculations in Fig. 6b and c), the incompletely random distribution of the microscopic polarization domains in the bulk polycrystalline samples fabricated using the spark plasma sintering technique under a uniaxial compression could allow a remanent macroscopic polarization and thus an effective piezoelectric response. Therefore, as a new kind of planar defects, the filling faults provide a new way to tune the physical properties and functionalities, including but not limited to electrical and piezoelectric properties, in crystalline solids.

## DISCUSSION

We report the finding of unique filling faults as a new kind of planar defects in Ni-based *XYZ*-type HH compounds, which induce the vacancy swap occupation of the *Y* atoms on both sides of the filling fault. This leads to the observation of a nearly equal occupation at 4c and 4d sites by the HAADF-STEM method, and is also consistent with the diffraction spectroscopy results that *Y* atoms occupy mainly at the 4c sites owing to the crystallographic equivalence of 4c and 4d sites in the HH lattice. When the *Y* element changes from Ni to Fe, the filling fault and associated vacancy swap occupation will disappear owing to the instability of the Fe-based Heusler phase. Whereas in the Co-based HH compounds, an incomplete filling fault tends to form. Furthermore, the filling faults and vacancy swap occupation can enhance the strain-induced polarization in HH compounds, and alter the carrier transport mechanism. Our findings deepen the understanding of precise crystallographic structures and defect–property relationships in solids and highlight that planar filling faults can elicit novel functionalities.

## Supplementary Material

nwag458_Supplemental_File

## Data Availability

Detailed materials and methods are provided in the online Supplementary data.
