# Supplementary material for: Filling faults and vacancy swap occupation in half-Heusler compounds
Source: Natl Sci Rev. 2026 Jul 27;13(18):nwag458. doi: 10.1093/nsr/nwag458 (PMC13596055; doi:10.1093/nsr/nwag458)
Supplement: Supplementary file 1 [file nwag458_supplemental_file.pdf]

Supplementary Information

Filling faults and vacancy swap occupation in half-Heusler compounds

Shen Han†, Zhongkang Han†, Yangjian Lin†, Ting Lin†, Tianqi Deng, Chenguang Fu*, Xiaojuan Hu, Fu Lv, Qizhu Li, Ziheng Gao, Haonan Sheng, Yi Huang, Mengzhao Chen, Pengfei Nan, Yuhui Huang, Binghui Ge, Lin Gu, G. Jeffrey Snyder, Tiejun Zhu*

Corresponding authors: chenguang_fu@zju.edu.cn; zhutj@zju.edu.cn

Materials and Methods

Synthesis

To ensure a side-by-side comparison, these five HH compounds and three nominal Heusler compounds were synthesized by the same method, i.e., levitation melting and spark plasma sintering. The ingots with nominal composition ZrNiSn, ScNiSb, TiCoSb, NbFeSb, VFeSb, $ZrNi_2Sn$, $TiCo_2Sb$, $NbFe_2Sb$, TiNiSn, HfNiSn, and LuNiSb were prepared by levitation melting of the stoichiometric amount of Sc (block, 99.9%), Lu (block, 99.9%), Ti (rod, 99.99%), Zr (rod, 99.99%), Hf (rod, 99.99%), V (foil, 99.5%), Nb (foil, 99.8%), Fe (block, 99.99%), Co (block, 99.99%), Ni (block, 99.99%), Sn (particle, 99.99%) and Sb (block, 99.999%) under an argon atmosphere for 3 minutes. Each ingot was remelted four times to ensure homogeneity. The ingots were ground manually into powders and mechanically milled (SPEX-8000D, PYNN Corporation) for 60 minutes under argon protection. The fine powders were compacted by SPS (LABOX-650F, Sinter Land Inc.) at 1123-1173 K under 65 MPa under vacuum for 10 minutes. The as-sintered VFeSb sample was annealed at 973K for 7 days to transfer the hexagonal phase to the cubic phase (*1*). The samples for (scanning) transmission electron microscopy ((S)TEM) characterization were prepared by traditional mechanical polishing, dimpling, and argon ion milling with a liquid nitrogen stage.

Material characterization and measurement

The phase structure of the sintered samples was evaluated by XRD on PANalytical Aries DY866 using Cu-K_α radiation (λ_0 = 1.5406 Å). The chemical compositions of all sintered samples were checked by the electron probe microanalyzer (EPMA, JEOL JXA-8100) with a wavelength dispersive spectroscope (WDS). The high-resolution HAADF, ABF images and SAED patterns were collected by using the Cs corrected JEOL ARM 200 F microscope at 200 kV. The room-temperature piezoelectric coefficient was determined through quasi-static force measurements employing a commercial PE coefficient meter (ZJ-3, IACAS), with a force magnitude of 0.25 N applied to the sample surfaces at a frequency of 110 Hz. Directions for the polarization and the applied force both align with the *Z*-axis for the measured polycrystalline ZrNiSn.

Energy-filtered multislice electron ptychography

MEP experimental data were acquired on a JEM-ARM300F2 transmission electron microscope equipped with Gatan K3 camera. A defocused electron probe positioned approximately 15 nm above the sample's surface scans across the specimen, and diffraction patterns at each scanned point are collected on a pixelated detector to form a 4D-STEM dataset with 200×200 diffraction patterns. The electron probe used a convergence semi-angle of 24.3 mrad

with a beam current of 10 pA. A scanning step size of approximately 0.524 Å and a dwell time of 0.8 ms were employed, resulting in an electron dose of 2×10^5 e/Å^2 that have negligible damage on the sample. To mitigate inelastic scattering effects, an energy slit with a 5 eV full width centered at zero-loss energy was applied during dataset acquisition, thereby enhancing the fidelity of the reconstructed MEP image. Reconstruction was performed through PtyRAD package (*2*), using a slice thickness of 1 nm. The sample thickness was approximately 35 nm in each region.

Atomic occupation calculations

All density functional theory (DFT) calculations were performed using the Vienna ab initio simulation package (VASP) (*3*, *4*) with the projector augmented wave (PAW) methods (*5*). The electron exchange-correlation interactions were described using the generalized gradient approximation (GGA) with the Perdew-Burke-Ernzerhof (PBE) functional (*6*). A plane-wave energy cutoff of 520 eV was employed, and a 2×3×3 gamma-point-centered k-point mesh was used. Geometry optimizations were carried out until the total energy differences and atomic force convergence criteria were below 10^{-5} eV and 0.02 eV/Å, respectively.

To simulate the distribution of Ni atoms in the 4c and 4d positions, $ZrNi_{1+\delta}Sn$ ($-0.25 \leq \delta \leq 0.25$) structures were randomly constructed with Ni atoms randomly placed in the 4c and 4d sites. The formation energy (E_f) of $ZrNi_{1+\delta}Sn$ was computed using the equation: $E_f = E(ZrNi_{1+\delta}Sn) - n\cdot\delta\cdot E(Ni) - E(ZrNiSn)$. $E(ZrNi_{1+\delta}Sn)$ is the total energy of the $ZrNi_{1+\delta}Sn$ system, n is the number of Zr or Sn atoms, E(Ni) is the energy of a single Ni atom, and E(ZrNiSn) is the energy of ZrNiSn.

Using the formation energies of $ZrNi_{1+\delta}Sn$ ($-0.25 \leq \delta \leq 0.25$) as training data, a cluster-expansion (CE) model was constructed iteratively with the assistance of the least absolute shrinkage and selection operator (LASSO) approach (*7*), as implemented in the CELL code (*8*). In total, 490 DFT-calculated $ZrNi_{1+\delta}Sn$ structures were used to refine the CE model. Canonical Metropolis Monte Carlo (MC) simulations were then performed to obtain the spatial distribution of Ni atoms.

In the CE model, the formation energy (E_s) for any configuration can be expressed as: $E_s = \sum_\alpha m_\alpha J_\alpha X_{s\alpha}$, where the summation is over all symmetrically inequivalent interactions (clusters) α, m_α represents the multiplicities (the number of symmetrically equivalent clusters), J_α are the effective cluster interactions (ECIs), and $X_{s\alpha}$ represents the cluster correlations, defined as the probability of finding cluster α in the configuration s. The ECIs are determined by fitting the predicted energies to the DFT-calculated energies of the training data set using the LASSO approach to avoid overfitting and to select the optimal cluster set.

Strain-induced polarization calculations

The strain-induced polarization is calculated with DFT using VASP (*3*, *4*). A small shear strain of 1% along the filling fault plane was applied to both the ideal ZrNiSn structure and the filling-fault contained structure. The strain-induced polarization was evaluated by computing the strain-induced charge density and electrostatic potential change. The planar average of the electrostatic potential change was performed for visualization.

Transport properties calculations

The electron-phonon, ionized-impurity, and grain-boundary scattering mechanisms were incorporated into the Boltzmann transport calculation using the state-of-the-art first-principles method implemented in EPW (*9*). The density functional (perturbation) theory calculations were performed using Quantum Espresso norm-conserving pseudopotentials from the PseudoDojo project, and a plane-wave cutoff energy of 90 Ry was employed for the wavefunctions. A 12×12×12 *k*-mesh was used for ground-state charge density and a coarse 8×8×8 *k*-/*q*-mesh for

Wannier-Fourier interpolation. The transport properties were computed using an interpolated 60×60×60 *k*-/*q*-mesh. The initial guess for Wannier functions includes *p* orbitals for Sn and *d* orbitals for Zr and Ni. Long-range interactions arising from Born effective charges were also considered. Dopant and free carrier screening effects were considered using the experimentally measured carrier concentrations (*10*).

The scattering due to complex planar defects like filling faults is beyond the current capability of first-principles Boltzmann transport theory, it is still possible to evaluate its contribution to the charge transport by considering it analogously as an interface between 4c and 4d domains. Thereafter, the generalized interfacial resistance can be evaluated using first-principles non-equilibrium Green's function method implemented in TranSIESTA (*11*, *12*). A filling fault structure was constructed between two semi-infinite electrodes with 4c and 4d configurations. A double-zeta plus polarization (DZP) basis was employed. The transmission was then computed for both filling-fault and defect-free structures to computed the interfacial resistance. We then estimated the density of such filling-faults using the amount of excess Ni (5%) and add its contribution to the conductivity from Boltzmann transport theory calculations for polycrystalline described in the previous paragraph

Piezoelectric sensor fabrication and testing

The sensors utilize polycrystalline TiNiSn, ZrNiSn, HfNiSn, ScNiSb, LuNiSb, and NbFeSb with dimensions of 3×3×1 mm^3. Copper sheets were pasted on the top and bottom surfaces of polycrystalline HH compounds using SAC305 solder. The output of piezoelectric voltage was measured by a programmable electrometer (Keithley 2182A) by finger tapping.

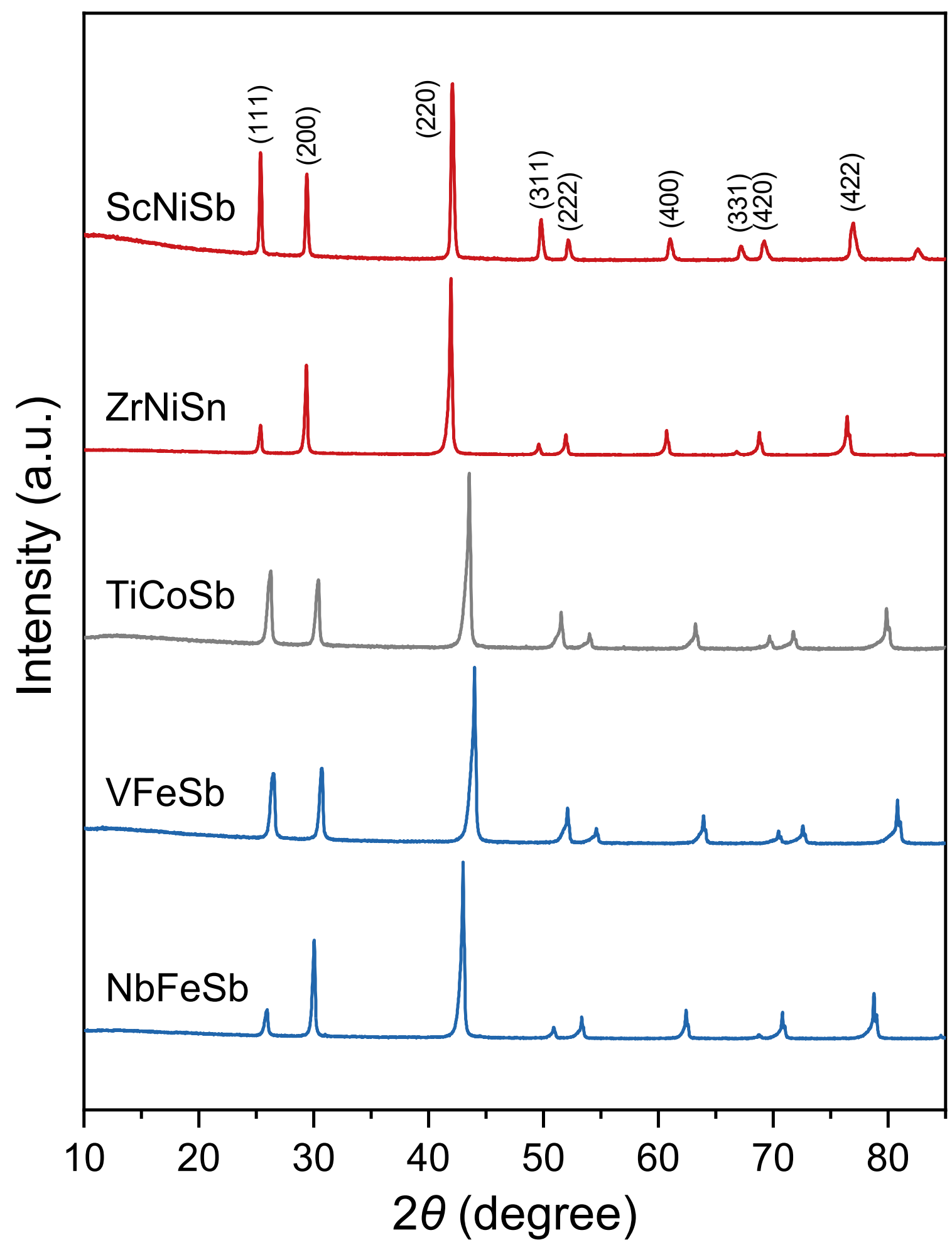

Fig. S1.
Room-temperature X-ray diffraction patterns for five selected HH compounds.

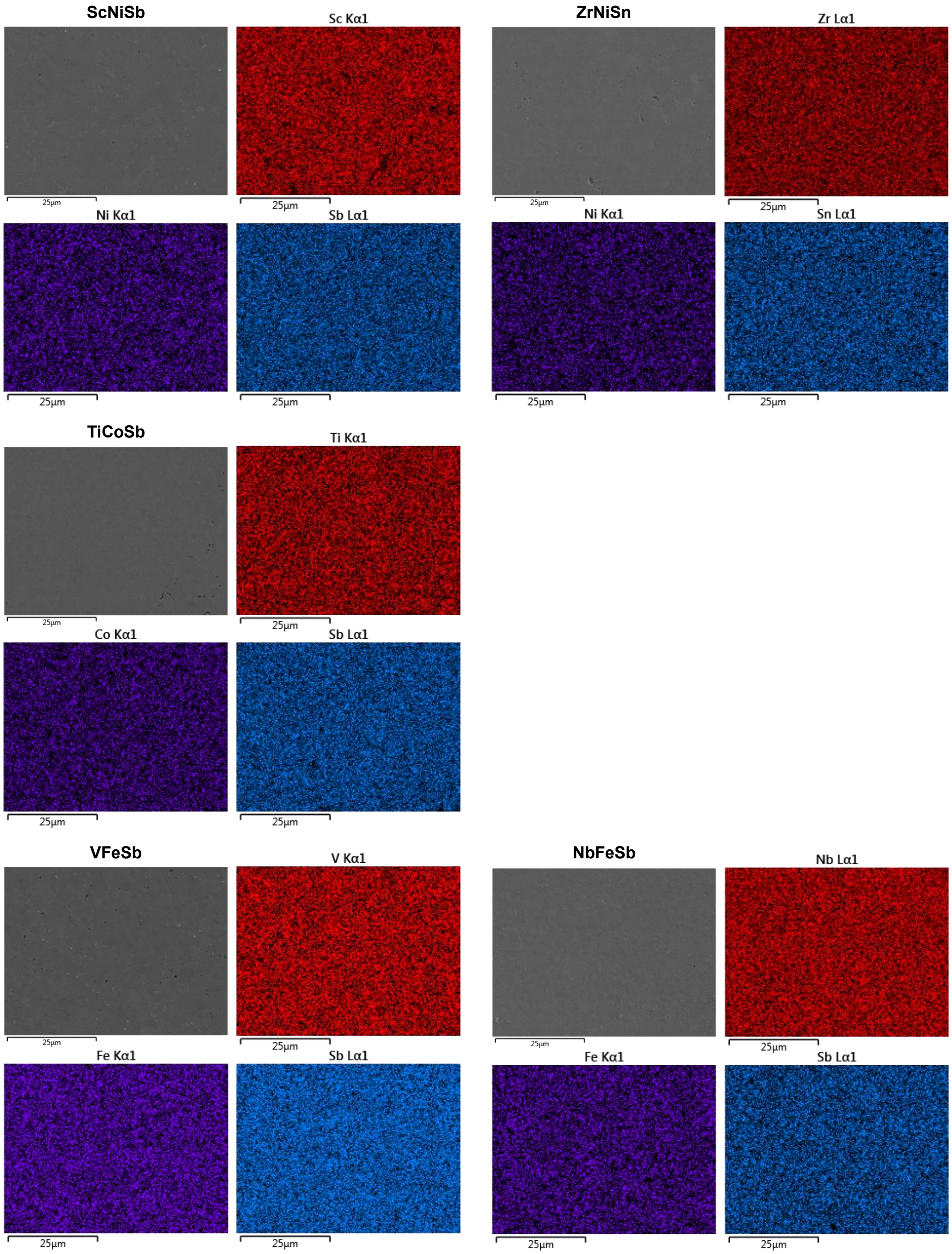

Fig. S2.
Energy-dispersive X-ray mapping for five selected HH compounds.

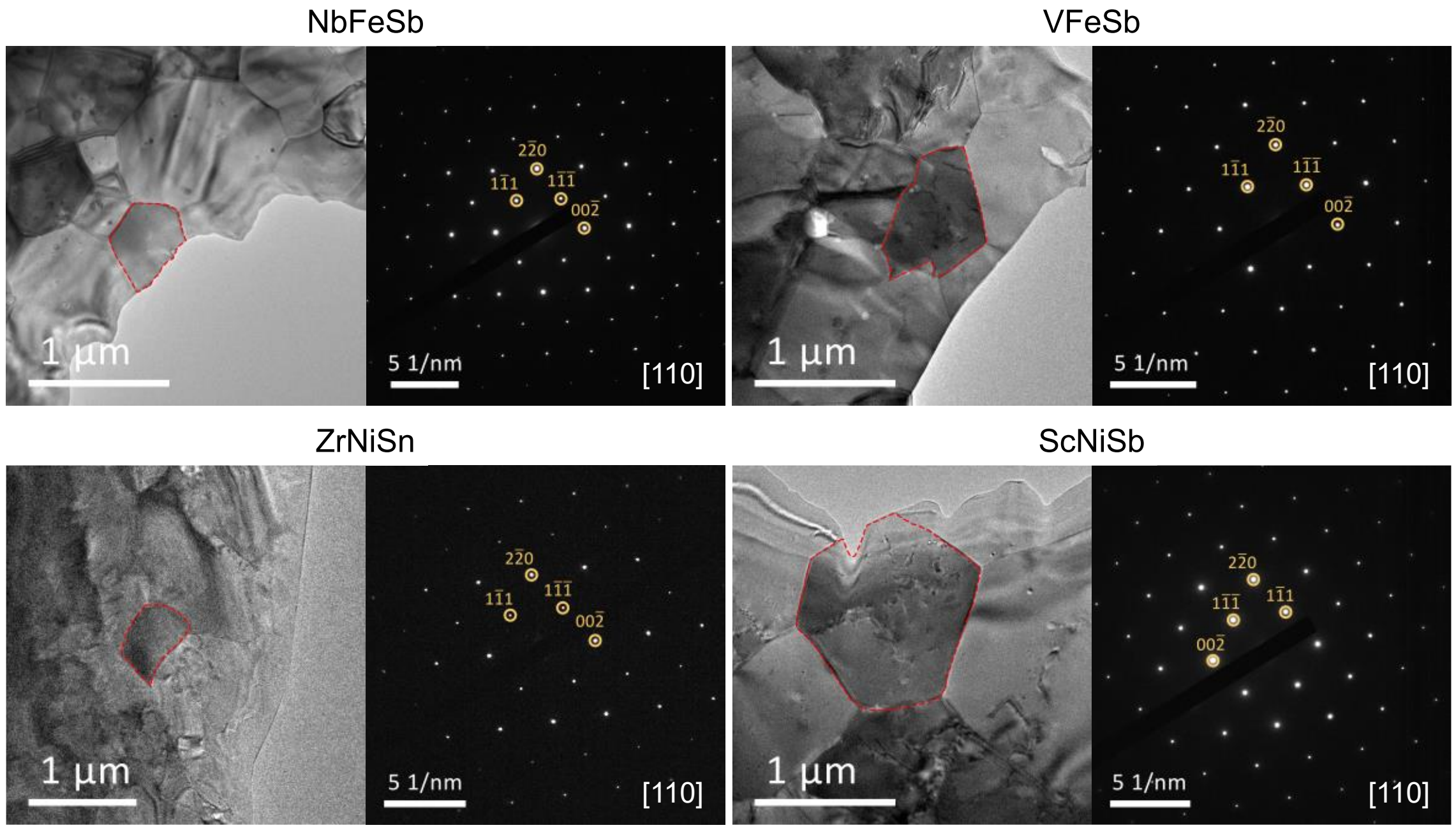

Fig. S3.

Low-magnification TEM images and selected area electron diffraction (SAED) patterns along [110] direction for NbFeSb, VFeSb, ZrNiSn, and ScNiSb. The selected grains are marked with red dashed lines.

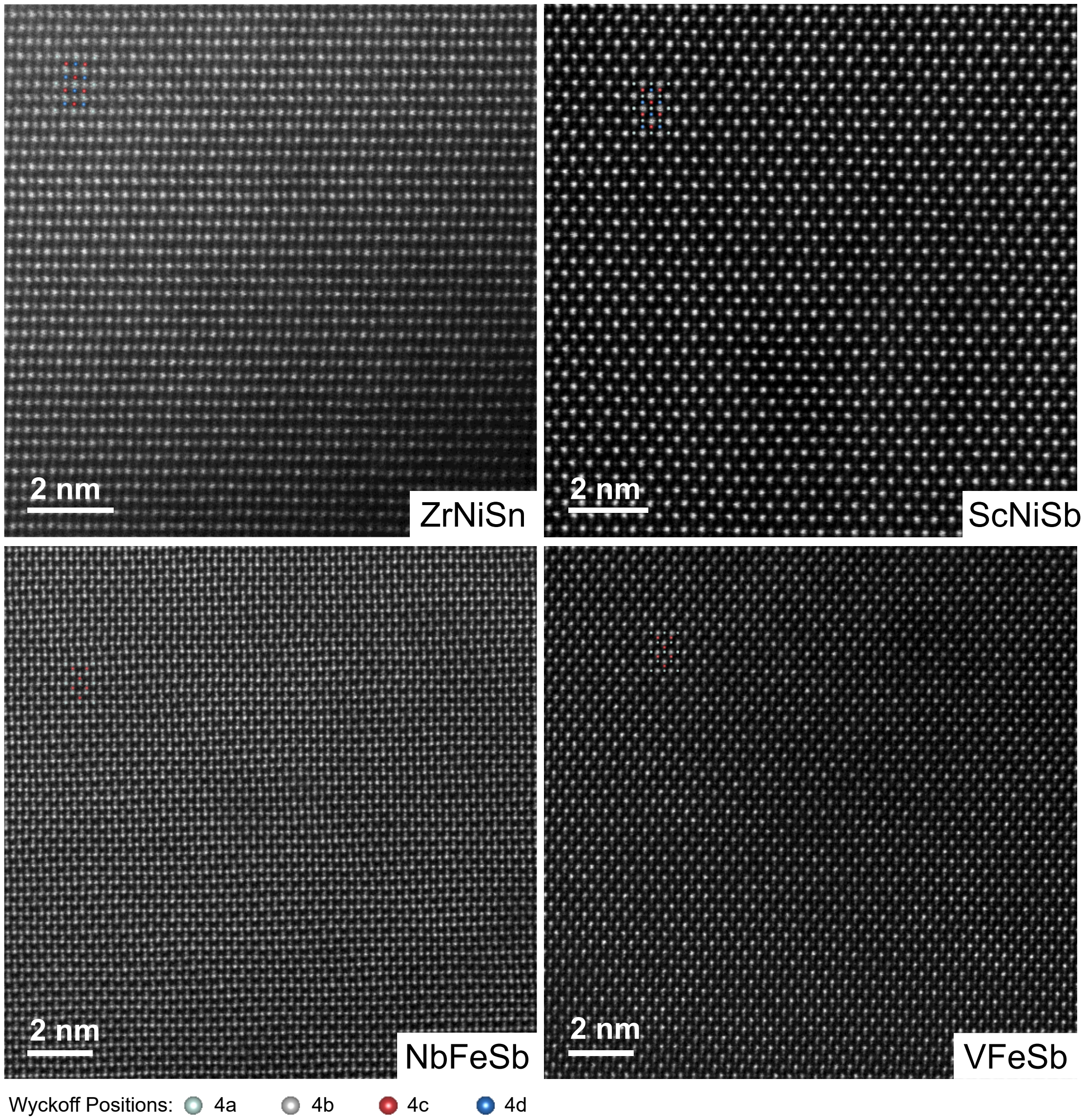

Fig. S4.

HAADF-STEM images along [110] direction in larger regions for ZrNiSn, ScNiSb, NbFeSb, and VFeSb. Ni atoms in ZrNiSn and ScNiSb show co-occupation in 4c and 4d sublattices, while Fe atoms in NbFeSb and VFeSb mainly occupy in 4c sublattice. Ball markers with different colors are used to distinguish different Wyckoff positions.

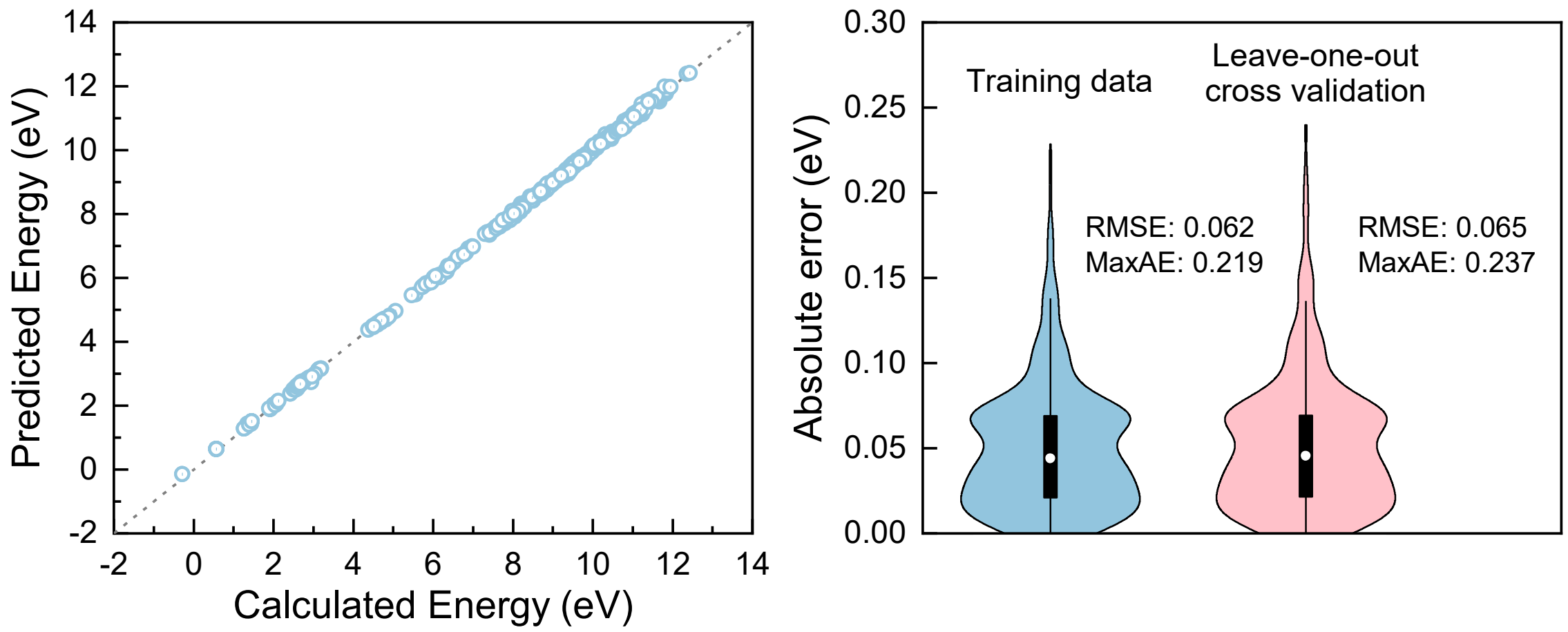

Fig. S5.

Cross-validation of predicted energies for CE approach in $ZrNi_{1+\delta}Sn$.

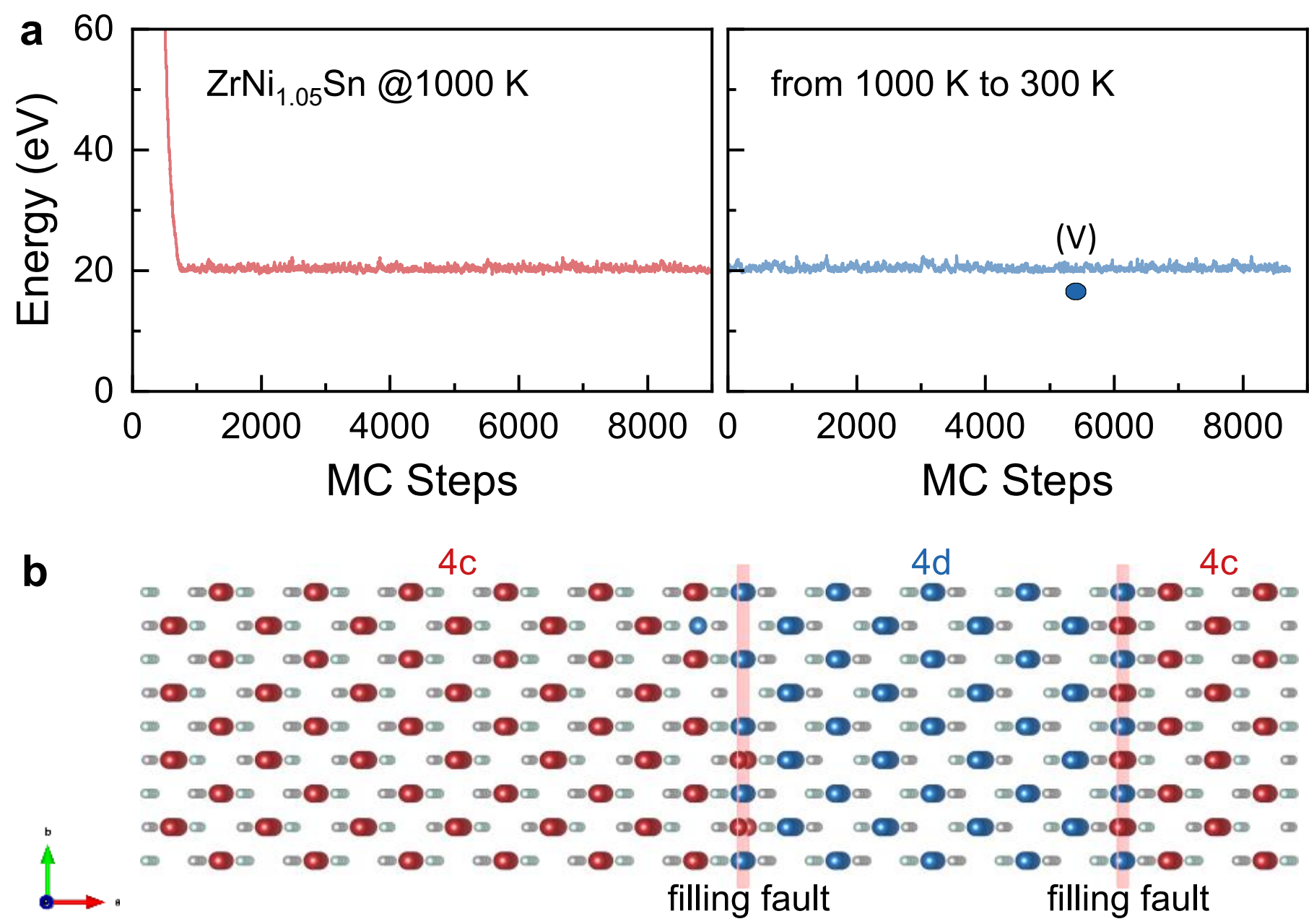

Fig. S6.

Monte-Carlo simulation for Ni occupancy in $ZrNi_{1.05}Sn$ from 1000 K to 300 K. (a) Formation energy changes of $ZrNi_{1.05}Sn$ from 1000 K to 300 K. (b) Atom occupations for stable structure (V) in (a).

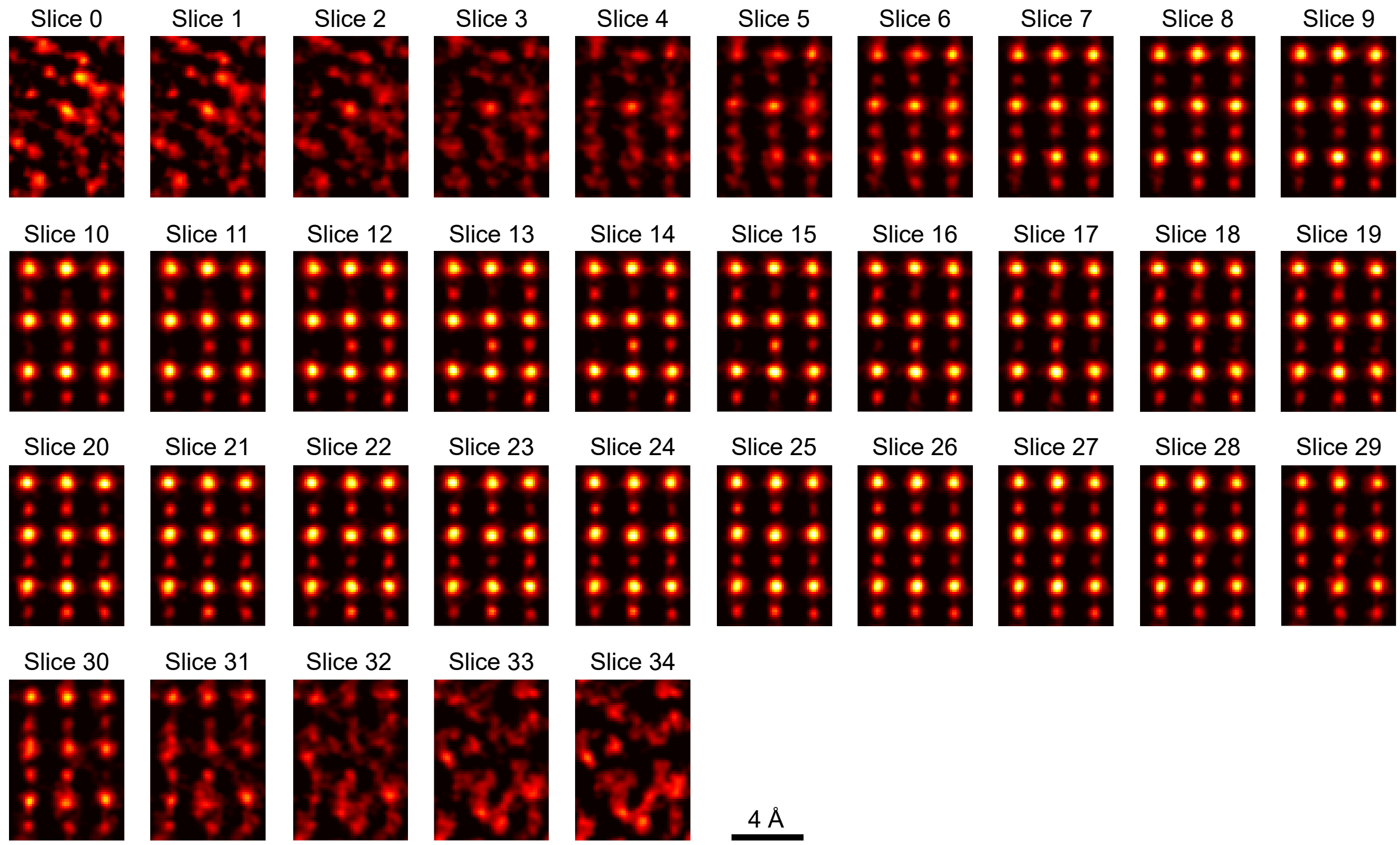

Fig. S7.
Full three-dimensional reconstructed phase images in ZrNiSn. Reconstruction results of all 35 slices for the presented region in Fig. 4b with depth = 1 nm for each individual slice.

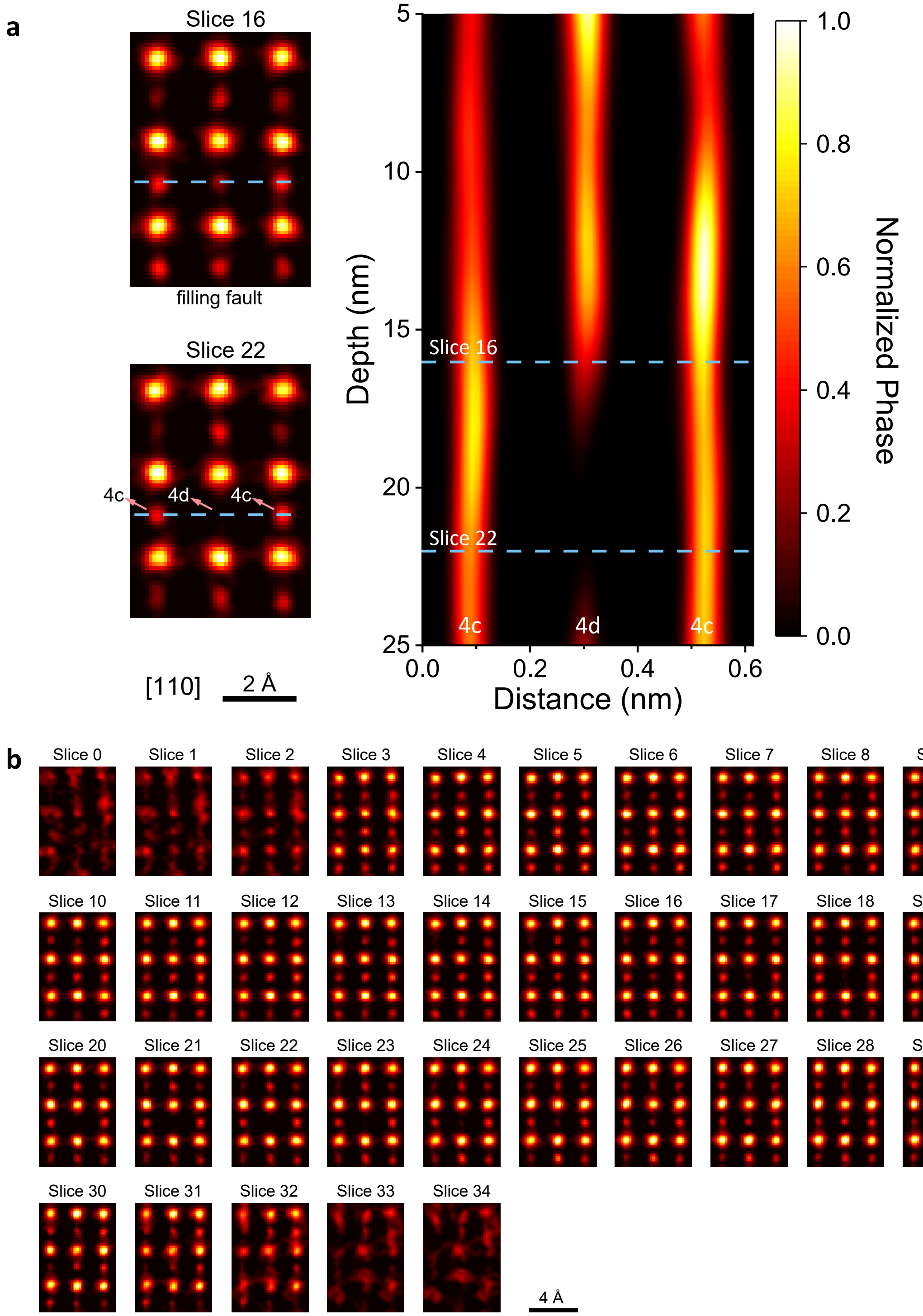

Fig. S8.

Full three-dimensional reconstructed results in ZrNiSn for anther region different from the one shown in Fig.4. (a) Phase image of slices along [110] direction. and depth profile along the dashed lines. (b) Reconstruction results of all 35 slices for the presented region in (a) with depth = 1 nm for each individual slice.

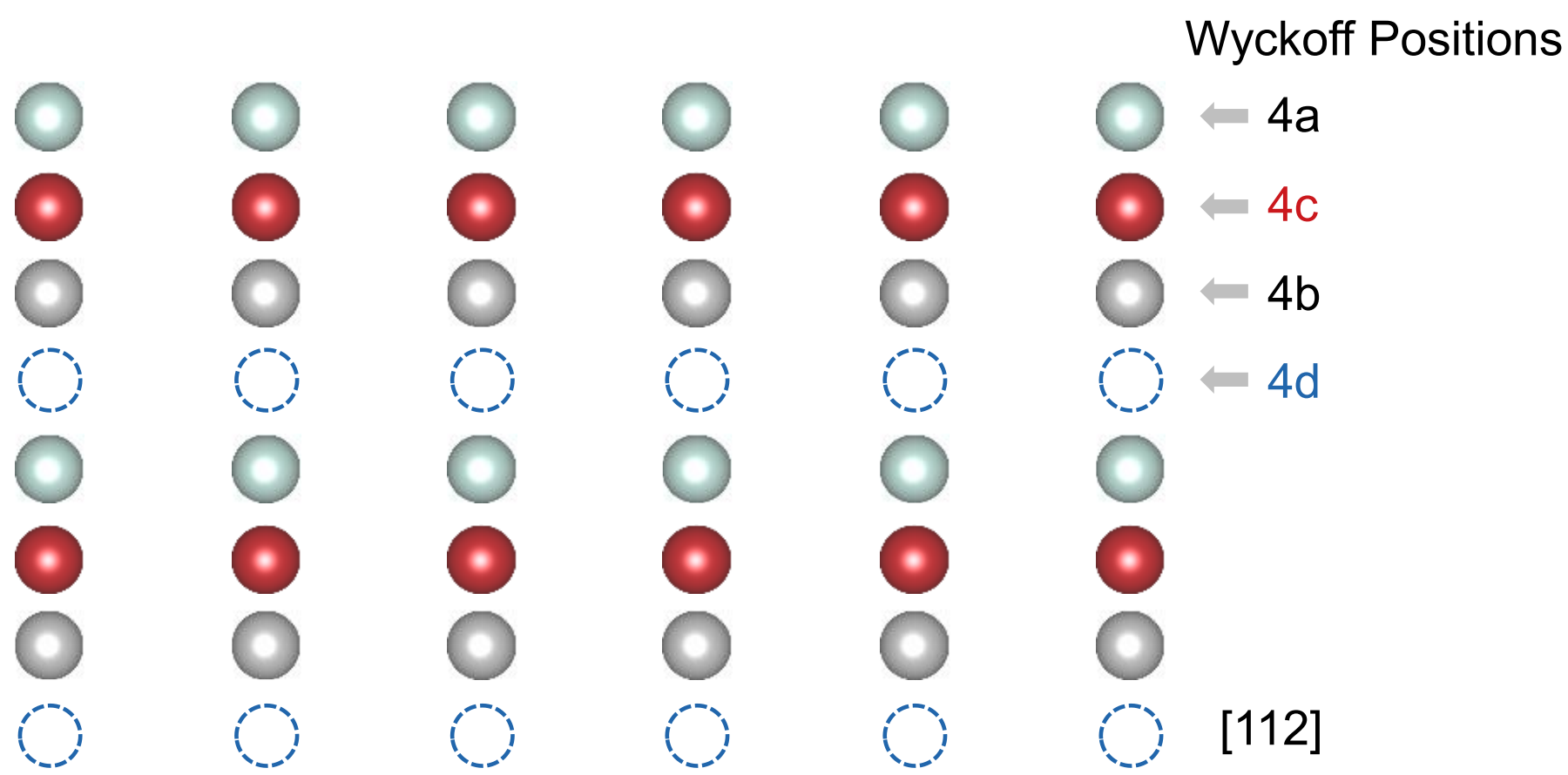

Fig. S9.

Schematic of atomic occupancy in the ideal half-Heusler structure along [112] direction. The four sets of sublattices separated at alternative planes.

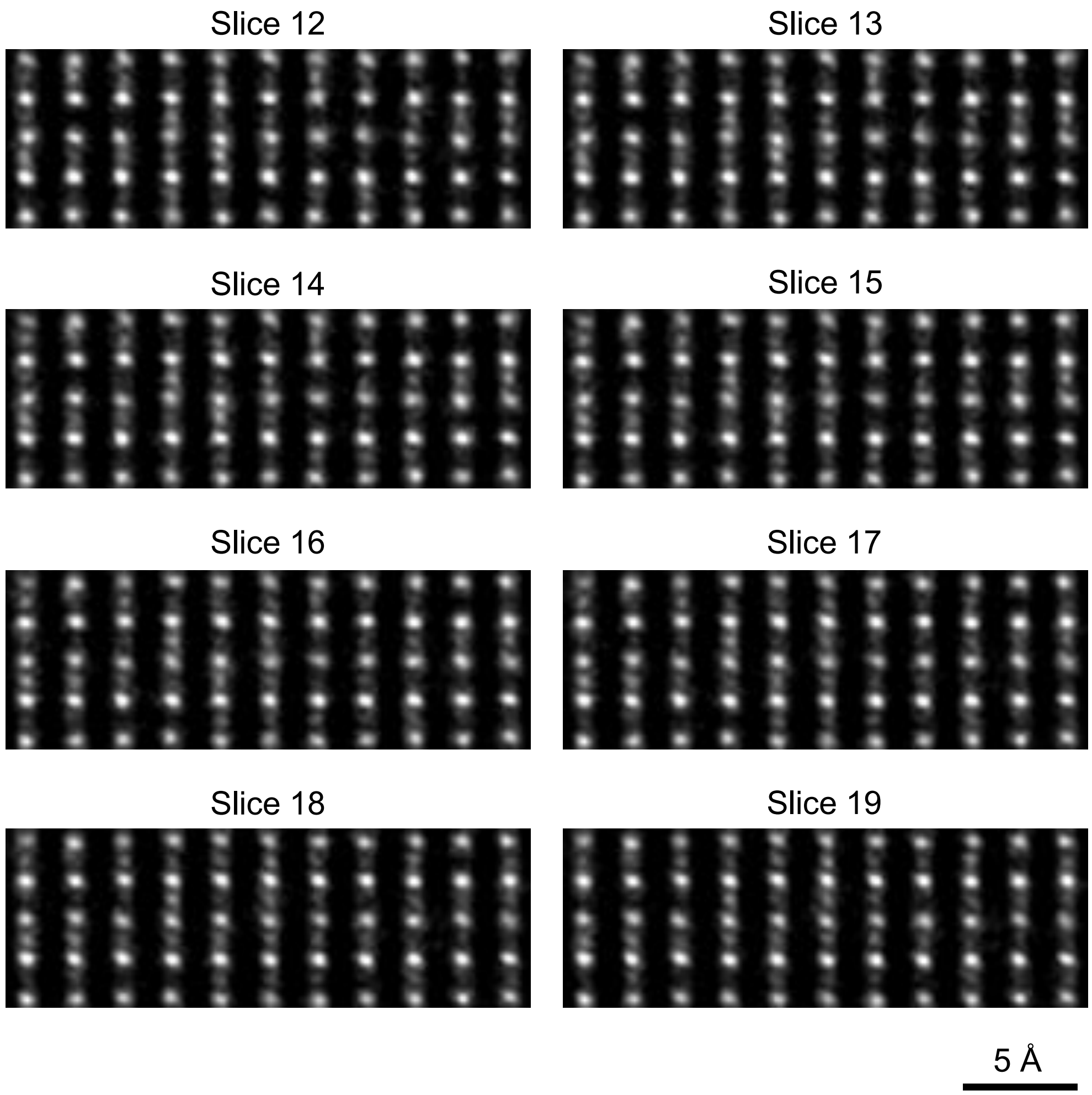

Fig. S10.
Phase image of slices in ZrNiSn along [112] direction.

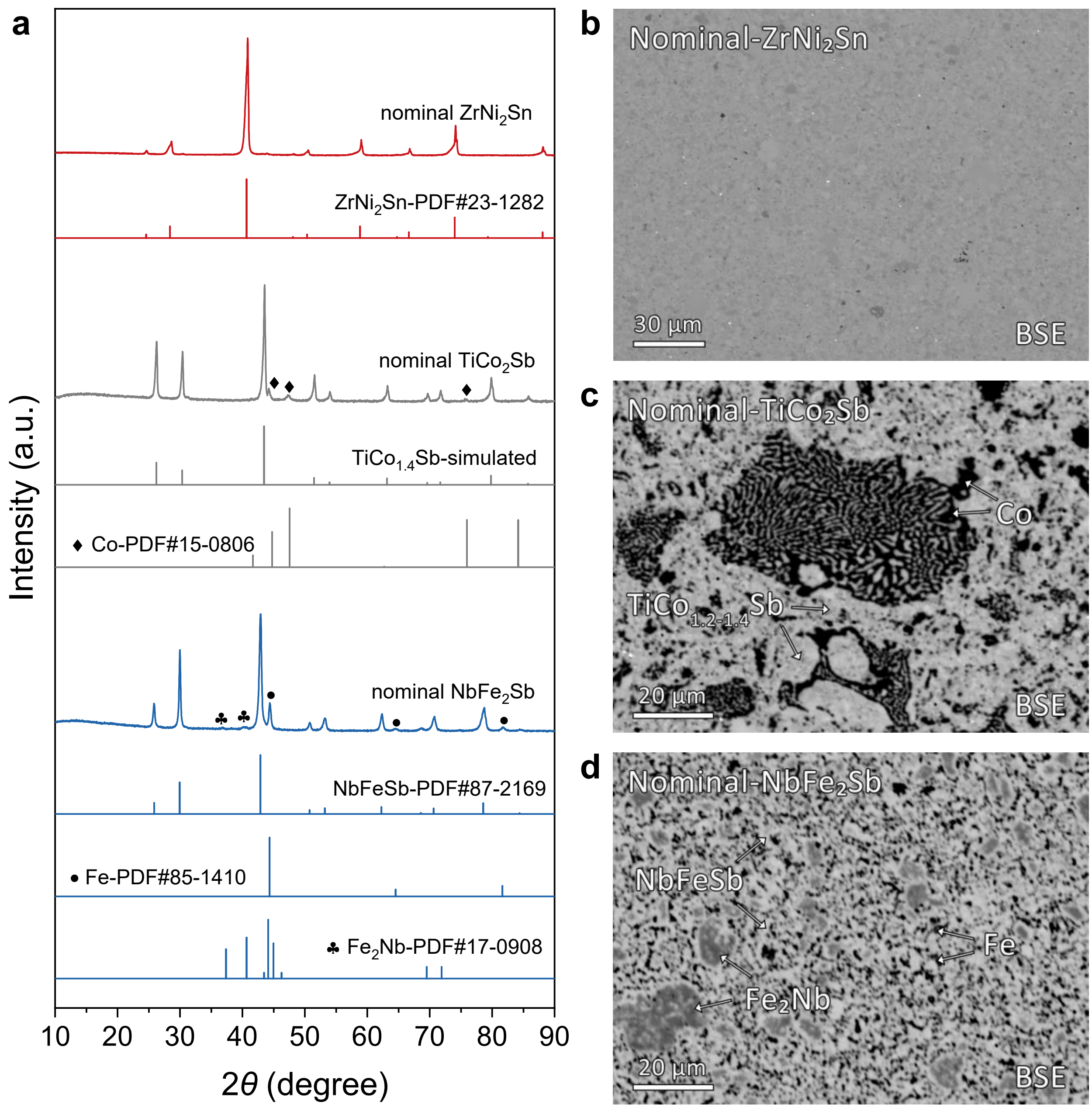

Fig. S11.

Phase and composition analysis of three nominal Heusler compounds. (a) Room-temperature X-ray diffraction patterns and (b-d) backscattered electron images for nominal $ZrNi_2Sn$, $TiCo_2Sb$, and $NbFe_2Sb$ samples.

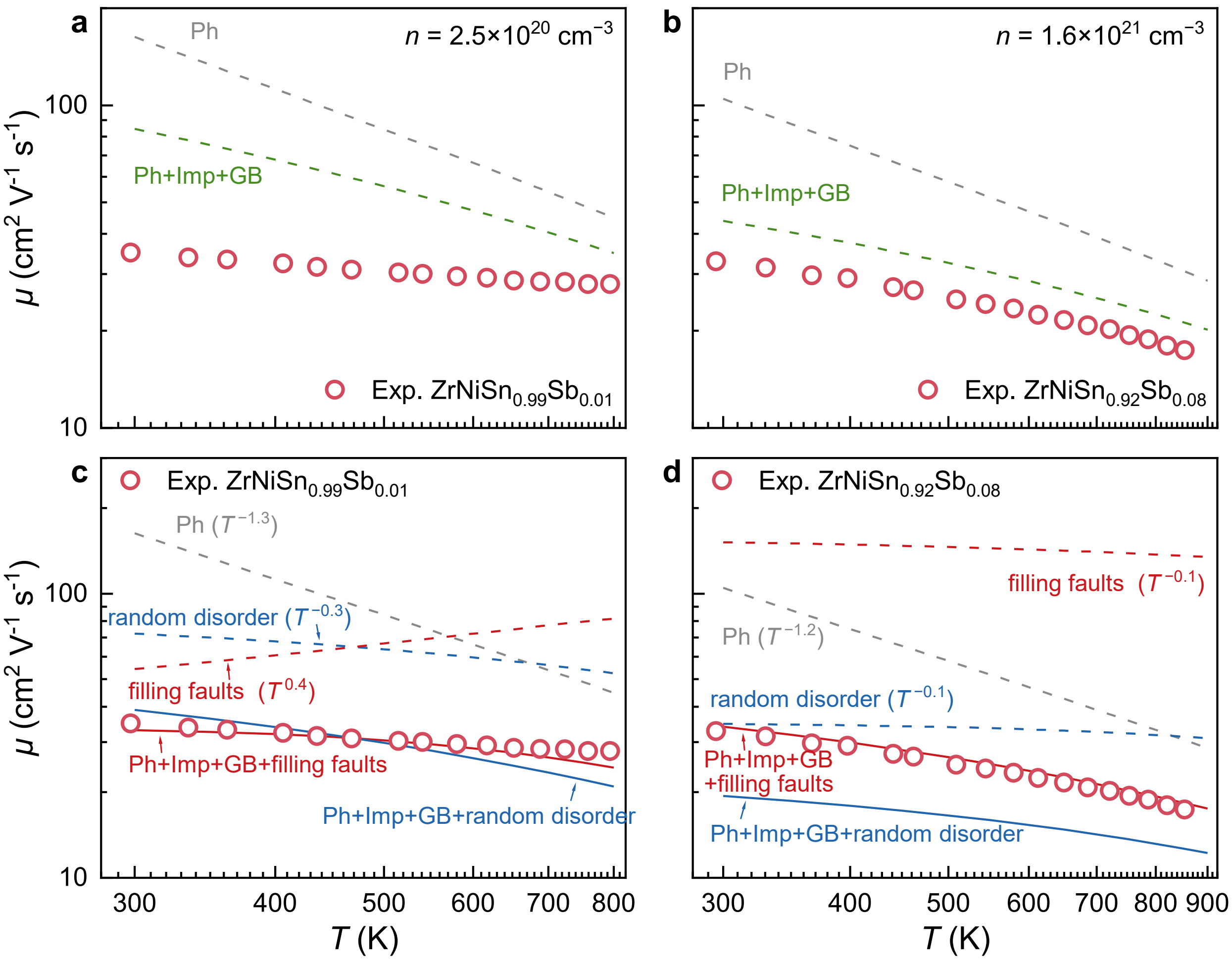

Fig. S12.

The comparison of the experimental and calculated temperature-dependent carrier mobility for ZrNiSn. (a and b) Only considering the scattering resources of electron-phonon (Ph) interaction, ionized-impurity (Imp), and grain boundary (GB). (c and d) Considering the additional effect of random disorder and filling faults, respectively.

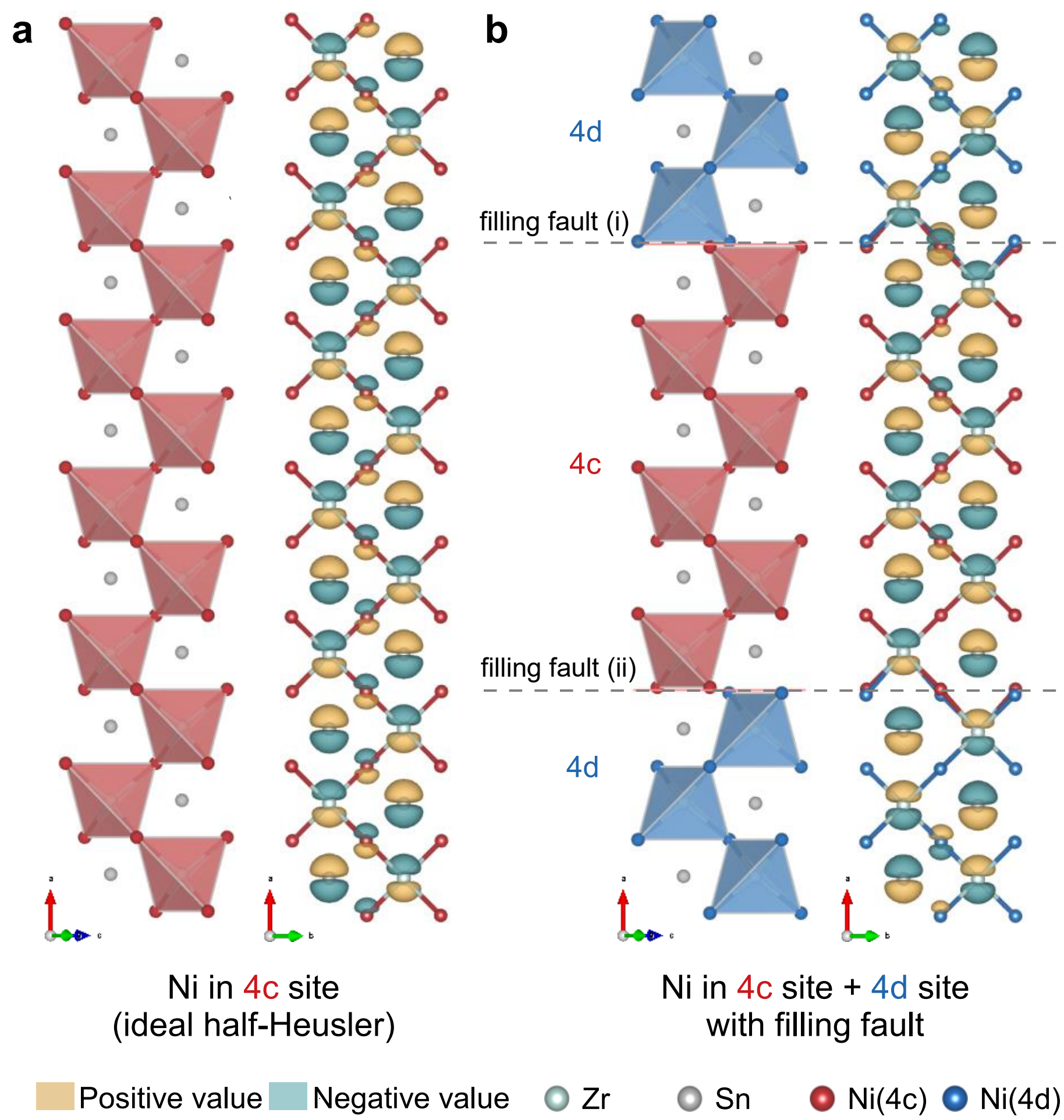

Fig. S13.

Calculation for strain-induced polarization in ZrNiSn. The crystal structure after relaxation and polarization charge distribution are provided in the ideal HH structure (a) and the structure with filling faults (b), respectively. The closed surface refers to the tetrahedral coordination of Zr atoms with the nearest neighboring Ni atoms in 4c (red) or 4d (blue) sites. The yellow and green shades correspond to positive and negative isosurface values of 0.002 e $bohr^{-3}$, respectively.

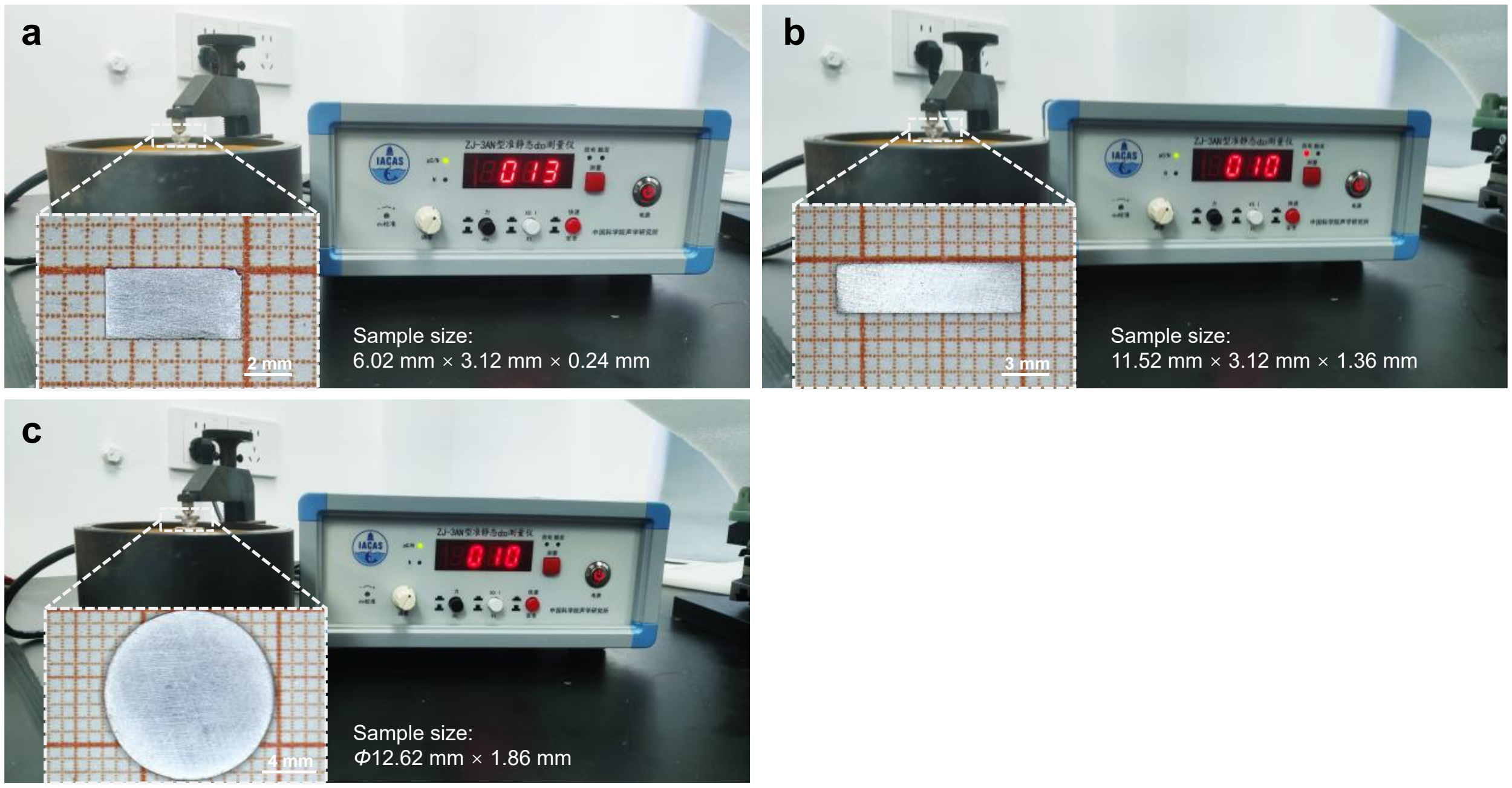

Fig. S14.

Measurement of piezoelectric coefficient in polycrystalline ZrNiSn. Samples with different sizes were used to comparatively examine the effect of thickness on piezoelectric coefficient d_{eff} measurement. (a) Thin slice (rectangle); (b) thick sheet (rectangle); (c) thick sheet (round).

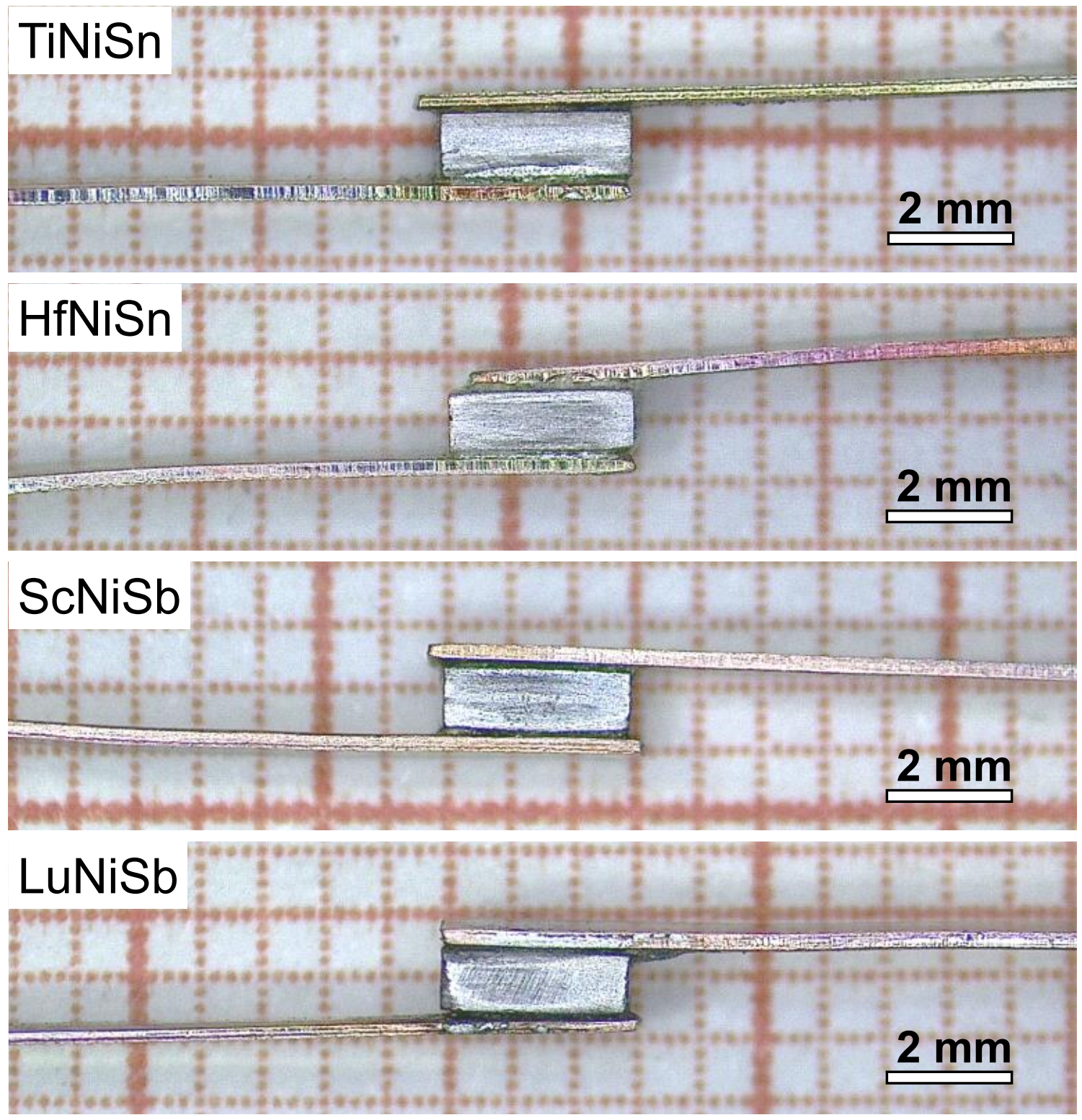

Fig. S15.
Optical images for piezoelectric sensors made by polycrystalline TiNiSn, HfNiSn, ScNiSb, and LuNiSb

Table S1.
Chemical composition analysis of the Fe-, Co-, and Ni-based HH compounds.

Nominal composition	Excess *Y*	Methods	Reference
ZrNiSn	5%	EPMA	(*13*)
ZrNiSn	1%	ICP	(*14*)
	3%	EDS	
ZrNiSn	3%	EPMA	(*15*)
HfNiSn	2%	EPMA	(*16*)
(Zr,Hf)NiSn	6%	EDS	(*17*)
ZrCoSb	2%	EDS	(*18*)
ZrCo(Sb,Sn)	3%	EDS	(*19*)
NbFeSb	2%	EPMA	(*20*)
TaFeSb	4%	EPMA	(*21*)

Table S2.
Occupation (Occ.) of *Y* in 4c and 4d sites of the Fe-, Co-, and Ni-based HH compounds with results from structural refinements based on diffraction spectroscopy methods.

Nominal composition	Occ. in 4c	Occ. in 4d	Methods	Reference
TiNiSn	100.0%	3.6%	NPD	(*22*)
TiNiSn	100.0%	2.1%	NPD	(*23*)
TiNiSn	100.0%	2.4%	NPD	(*24*)
ZrNiSn	95.3%	8.5%	SR-PXRD	(*25*)
ZrNiSn	100.0%	4.6%	SR-PXRD	(*13*)
ZrNiSn	100.0%	2.2%	PXRD	(*16*)
HfCoSb	100%	2.3%	PXRD	(*26*)
ZrCo(Sb,Sn)	100%	1.9%	PXRD	(*19*)
VFeSb	91.0%	8.0%	SR-PXRD	(*27*)
VFeSb	100%	6.4%	SR-PXRD	(*28*)
VFeSb	95.0%	5.0%	PXRD	(*29*)
NbFeSb	100%	0.9%	PXRD	(*30*)
Ti(Fe,Cu)Sb	100%	8.0%	PXRD	(*31*)

Table S3.

Occupation (Occ.) of *Y* in 4c and 4d sites of the Fe-, Co-, and Ni-based HH compounds with results from HAADF-STEM observations. The data comes from the intensity profile graph, where multiple points are taken and averaged.

Nominal composition	Occ. in 4c	Occ. in 4d	Reference
(Ti,Hf)NiSn	54.8%	45.2%	(*32*)
(Ti,Hf)NiSn	53.6%	46.4%	(*33*)
ZrNiSn	50.1%	49.9%	(*15*)
ZrCo(Sb,Sn)	53.8%	46.2%	(*19*)
HfCoSb	60.4%	39.6%	(*26*)
(Nb,Hf)FeSb	88.1%	11.9%	(*34*)
Ti(Fe,Cu)Sb	90.9%	9.1%	(*31*)

Table S4.
The detailed EPMA composition results of five selected HH compounds.

Position No.		Atomic ratio (%)	
	**Zr**	**Ni**	**Sn**
1	31.59	34.37	34.05
2	32.12	34.16	33.73
3	30.98	36.51	32.51
4	30.84	35.88	33.28
average	31.38	35.23	33.39
	**Sc**	**Ni**	**Sb**
1	37.51	31.05	31.44
2	37.22	31.13	31.66
3	37.58	30.01	32.41
4	37.65	29.69	32.66
average	37.49	30.47	32.04
	**Ti**	**Co**	**Sb**
1	34.17	33.35	32.48
2	34.19	33.40	32.41
3	34.62	33.21	32.16
4	34.53	33.29	32.18
average	34.38	33.31	32.31
	**Nb**	**Fe**	**Sb**
1	32.69	34.31	32.98
2	32.30	34.35	33.35
3	32.62	34.20	33.17
4	32.47	34.13	33.40
average	32.52	34.25	33.23
	**V**	**Fe**	**Sb**
1	34.06	33.59	32.35
2	34.56	33.41	32.02
3	33.96	33.32	32.72
4	33.50	33.37	33.13
average	34.02	33.42	32.56

Table S5.
The normalized EPMA composition and relative density of five selected HH compounds.

Nominal composition	EPMA composition	Relative density
ZrNiSn	$Zr_{0.94}Ni_{1.06}Sn$	99.9%
ScNiSb	$Sc_{1.17}Ni_{0.95}Sb$	98.7%
TiCoSb	$Ti_{1.06}Co_{1.03}Sb$	98.6%
NbFeSb	$Nb_{0.98}Fe_{1.03}Sb$	99.2%
VFeSb	$V_{1.04}Fe_{1.03}Sb$	97.9%

Table S6.
Formation enthalpy of Fe/Co/Ni-based HH and Heusler compounds.

	Formation enthalpy ΔH_f (eV/atom)	
	DFT calculations in this work	OQMD database (*35*, *36*)
ZrNiSn	-0.715	-0.719
$ZrNi_2Sn$	-0.527	-0.534
NbFeSb	-0.359	-0.350
$NbFe_2Sb$	-0.029	-0.023
TiCoSb	-0.663	-0.681
$TiCo_2Sb$	-0.272	-0.292
$TiCo_{1.5}Sb$	-0.403	-0.428

Supplementary Note 1

Stability and synthesis of corresponding Heusler compounds

Three samples with nominal Heusler compositions, $ZrNi_2Sn$, $TiCo_2Sb$, and $NbFe_2Sb$, were synthesized via the same method as the polycrystalline HH samples (Materials and Methods: Synthesis) to examine their stability.

No obvious impurity phase was observed in the XRD pattern (Fig. S11a) and backscattered electron (BSE) image (Fig. S11b) of the $ZrNi_2Sn$ sample, suggesting that this composition can be stabilized under the same synthesis conditions as ZrNiSn. However, obvious impurity phases were observed in the nominal compositions $TiCo_2Sb$ and $NbFe_2Sb$. In the BSE image of nominal $TiCo_2Sb$ (Fig. S11c), the grey matrix exhibits an actual composition of $TiCo_{1.2-1.4}Sb$, with excess Co-precipitations appearing as the other black components. The simulated XRD pattern of $TiCo_{1.4}Sb$ by VESTA software is consistent with the main phase in the experimental XRD result for the nominal $TiCo_2Sb$ sample, in which the remaining impurity phase was identified as Co (Fig. S11a). The main phase of the nominal $NbFe_2Sb$ is NbFeSb along with the impurity phases as Fe and Fe_2Nb (Fig. S11a and d).

Supplementary Note 2

Stability and synthesis of corresponding Heusler compounds

As planar defects, filling faults may potentially affect the carrier transport. After clarifying their structural features, we attempted to quantitatively analyze their scattering strength using first-principles calculations. For the first step, the electron-phonon (Ph) interaction, ionized-impurity (Imp) scattering from dopants, and grain-boundary (GB) scattering are considered for doped polycrystalline ZrNiSn compounds, which can be evaluated fully from first principles within the Boltzmann transport theory. Experimental data from ZrNiSn compositions with different carrier concentrations (*10*) were used for comparison with the calculations. As shown in Figs. S12a and S12b, the calculated carrier mobility based on these three scattering resources show an overestimation relative to the experimental data, indicating that additional scattering mechanisms are operative.

Next, we compare two forms of additional scattering resources. For the real composition of ZrNiSn, identified using EPMA, there is an excess of Ni of around 5%. In one scenario, these excess Ni atoms occupy the vacant 4d sites by forming the random disorder. In another scenario, we assume that these excess Ni atoms form the filling faults. While the scattering due to filling faults is beyond the current capability of first-principles Boltzmann transport theory, it is still possible to evaluate its contribution to the charge transport by considering it analogously as an interface between 4c and 4d domains. Thereafter, the generalized interfacial resistance can be evaluated using first-principles calculations, which can be evaluated using first-principles non-equilibrium Green's function method (Methods: Transport properties calculations). This allows for a quantitative comparison of their respective scattering strengths.

After incorporating the above two scattering mechanisms, the comparison between the calculated mobility values and the experimental data is presented in Fig. S12c and d. For the low carrier concentration composition (Fig. S12c), the scattering strength induced by Ni random disorder is comparable to that of filling faults. However, its mobility exhibits a negative temperature dependence $T^{-0.3}$, while filling faults presents a positive one, i.e., $T^{0.4}$. Consequently, the experimental data fails to align with the calculated total mobility when random disorder is included but matches well the calculated one when filling faults is considered. For the higher carrier concentration composition (Fig. S12d), due to the screening effect, both random disorder and filling faults yield a mobility temperature dependence approaching $T^{-0.1}$. Nevertheless, the

scattering strength from random disorder remains very large under these conditions, resulting in a calculated total mobility much lower than the experimental data. When filling faults are included instead, the calculated mobility for the high-carrier-concentration composition still agrees well with the experimental data. Based on the quantitatively comparative analysis of the calculations and experimental data in the two ZrNiSn compositions with different carrier concentrations, the influence of random disorder can be ruled out while the actual scattering effect should occur in the form of filling faults.

References

1. C. Fu, H. Xie, Y. Liu, T. J. Zhu, J. Xie, X. B. Zhao, Thermoelectric properties of FeVSb half-Heusler compounds by levitation melting and spark plasma sintering. *Intermetallics* **32**, 39–43 (2013).
2. C.-H. Lee, S. E. Zeltmann, D. Yoon, D. Ma, D. A. Muller, PtyRAD: A High-performance and Flexible Ptychographic Reconstruction Framework with Automatic Differentiation. *Microsc. Microanal.* **31**, 66–68 (2025).
3. G. Kresse, J. Hafner, Ab initio molecular dynamics for open-shell transition metals. *Phys. Rev. B* **48**, 13115–13118 (1993).
4. G. Kresse, J. Furthmüller, Efficient iterative schemes for ab initio total-energy calculations using a plane-wave basis set. *Phys. Rev. B* **54**, 11169–11186 (1996).
5. G. Kresse, D. Joubert, From ultrasoft pseudopotentials to the projector augmented-wave method. *Phys. Rev. B* **59**, 1758–1775 (1999).
6. J. P. Perdew, K. Burke, M. Ernzerhof, Generalized Gradient Approximation Made Simple. *Phys. Rev. Lett.* **77**, 3865–3868 (1996).
7. L. Barroso-Luque, P. Zhong, J. H. Yang, F. Xie, T. Chen, B. Ouyang, G. Ceder, Cluster expansions of multicomponent ionic materials: Formalism and methodology. *Phys. Rev. B* **106**, 144202 (2022).
8. See http://sol.physik.hu-berlin.de/cell for the documentation, tutorials, and installation instructions of the CELL code.
9. H. Lee, S. Poncé, K. Bushick, S. Hajinazar, J. Lafuente-Bartolome, J. Leveillee, C. Lian, J.-M. Lihm, F. Macheda, H. Mori, H. Paudyal, W. H. Sio, S. Tiwari, M. Zacharias, X. Zhang, N. Bonini, E. Kioupakis, E. R. Margine, F. Giustino, Electron–phonon physics from first principles using the EPW code. *npj Comput. Mater.* **9**, 156 (2023).
10. H. Xie, H. Wang, C. Fu, Y. Liu, G. J. Snyder, X. Zhao, T. Zhu, The intrinsic disorder related alloy scattering in ZrNiSn half-Heusler thermoelectric materials. *Sci. Rep.* **4**, 6888 (2015).
11. A. García, N. Papior, A. Akhtar, E. Artacho, V. Blum, E. Bosoni, P. Brandimarte, M. Brandbyge, J. I. Cerdá, F. Corsetti, R. Cuadrado, V. Dikan, J. Ferrer, J. Gale, P. García-Fernández, V. M. García-Suárez, S. García, G. Huhs, S. Illera, R. Korytár, P. Koval, I. Lebedeva, L. Lin, P. López-Tarifa, S. G. Mayo, S. Mohr, P. Ordejón, A. Postnikov, Y. Pouillon, M. Pruneda, R. Robles, D. Sánchez-Portal, J. M. Soler, R. Ullah, V. W. Yu, J. Junquera, S_{iesta}: Recent developments and applications. *J. Chem. Phys.* **152**, 204108 (2020).
12. N. Papior, N. Lorente, T. Frederiksen, A. García, M. Brandbyge, Improvements on non-equilibrium and transport Green function techniques: The next-generation transiesta. *Comput. Phys. Commun.* **212**, 8–24 (2017).
13. H. H. Xie, J. L. Mi, L. P. Hu, N. Lock, M. Chirstensen, C. G. Fu, B. B. Iversen, X. B. Zhao, T. J. Zhu, Interrelation between atomic switching disorder and thermoelectric properties of ZrNiSn half-Heusler compounds. *CrystEngComm* **14**, 4467–4471 (2012).
14. C. Fu, M. Yao, X. Chen, L. Z. Maulana, X. Li, J. Yang, K. Imasato, F. Zhu, G. Li, G. Auffermann, U. Burkhardt, W. Schnelle, J. Zhou, T. Zhu, X. Zhao, M. Shi, M. Dressel, A. V. Pronin, G. J. Snyder, C. Felser, Revealing the Intrinsic Electronic Structure of 3D Half-Heusler Thermoelectric Materials by Angle-Resolved Photoemission Spectroscopy. *Adv. Sci.* **7**, 1902409 (2020).

15. Z. Li, W. Xue, S. Han, Z. Gao, A. Li, X. Zhao, Y. Wang, C. Fu, T. Zhu, Ni atomic disorder in ZrNiSn revealed by scanning transmission electron microscopy. *Mater. Today Phys.* **34**, 101072 (2023).
16. V. V. Romaka, P. Rogl, L. Romaka, Y. Stadnyk, A. Grytsiv, O. Lakh, V. Krayovskii, Peculiarities of structural disorder in Zr- and Hf-containing Heusler and half-Heusler stannides. *Intermetallics* **35**, 45–52 (2013).
17. K. Gałązka, W. Xie, S. Populoh, M. H. Aguirre, S. Yoon, G. Büttner, A. Weidenkaff, Tailoring thermoelectric properties of $Zr_{0.43}Hf_{0.57}NiSn$ half-Heusler compound by defect engineering. *Rare Met.* **39**, 659–670 (2020).
18. R. He, H. Zhu, J. Sun, J. Mao, H. Reith, S. Chen, G. Schierning, K. Nielsch, Z. Ren, Improved thermoelectric performance of n-type half-Heusler $MCo_{1-x}Ni_xSb$ (M = Hf, Zr). *Mater. Today Phys.* **1**, 24–30 (2017).
19. R. He, T. Zhu, Y. Wang, U. Wolff, J.-C. Jaud, A. Sotnikov, P. Potapov, D. Wolf, P. Ying, M. Wood, Z. Liu, L. Feng, N. P. Rodriguez, G. J. Snyder, J. C. Grossman, K. Nielsch, G. Schierning, Unveiling the phonon scattering mechanisms in half-Heusler thermoelectric compounds. *Energy Environ. Sci.* **13**, 5165–5176 (2020).
20. C. Fu, T. Zhu, Y. Liu, H. Xie, X. Zhao, Band engineering of high performance p-type FeNbSb based half-Heusler thermoelectric materials for figure of merit zT > 1. *Energy Environ. Sci.* **8**, 216–220 (2015).
21. A. Grytsiv, V. V. Romaka, N. Watson, G. Rogl, H. Michor, B. Hinterleitner, S. Puchegger, E. Bauer, P. Rogl, Thermoelectric Half-Heusler compounds TaFeSb and $Ta_{1-x}Ti_xFeSb$ ($0 \leq x \leq 0.11$): Formation and physical properties. *Intermetallics* **111**, 106468 (2019).
22. R. A. Downie, D. A. MacLaren, R. I. Smith, J. W. G. Bos, Enhanced thermoelectric performance in tinisn-based half-Heuslers. *Chem. Commun.* **49**, 4184–4186 (2013).
23. R. A. Downie, R. I. Smith, D. A. MacLaren, J.-W. G. W. G. Bos, Metal Distributions, Efficient n-Type Doping, and Evidence for in-Gap States in $TiNiM_ySn$ (M = Co, Ni, Cu) half-Heusler Nanocomposites. *Chem. Mater.* **27**, 2449–2459 (2015).
24. R. A. Downie, S. A. Barczak, R. I. Smith, J. W. G. Bos, Compositions and thermoelectric properties of XNiSn (X = Ti, Zr, Hf) half-Heusler alloys. *J. Mater. Chem. C* **3**, 10534–10542 (2015).
25. H. Miyazaki, T. Nakano, M. Inukai, K. Soda, Y. Izumi, T. Muro, J. Kim, M. Takata, M. Matsunami, S. I. Kimura, Y. Nishino, Electronic and local crystal structures of the ZrNiSn Half-Heusler thermoelectric material. *Mater. Trans.* **55**, 1209–1214 (2014).
26. R. He, T. Zhu, P. Ying, J. Chen, L. Giebeler, U. Kühn, J. C. Grossman, Y. Wang, K. Nielsch, High-Pressure-Sintering-Induced Microstructural Engineering for an Ultimate Phonon Scattering of Thermoelectric Half-Heusler Compounds. *Small* **17**, 2102045 (2021).
27. Y. Huang, K. Hayashi, Y. Miyazaki, Electron Conduction Mechanism of Deficient Half-Heusler VFeSb Compound Revealed by Crystal and Electronic Structure Analyses. *Chem. Mater.* **32**, 5173–5181 (2020).
28. D. A. Ferluccio, B. F. Kennedy, S. A. Barczak, S. R. Popuri, C. Murray, M. Pollet, J.-W. G. Bos, Thermal properties of TiNiSn and VFeSb half-Heusler thermoelectrics from synchrotron x-ray powder diffraction. *J. Phys. Energy* **3**, 035001 (2021).

29. N. S. Chauhan, Y. Miyazaki, A mechanistic view of defect engineered VFeSb half-Heusler alloys. *Mater. Adv.* **3**, 8498–8511 (2022).
30. D. Hobbis, R. P. Hermann, H. Wang, D. S. Parker, T. Pandey, J. Martin, K. Page, G. S. Nolas, Structural, Chemical, Electrical, and Thermal Properties of n -Type NbFeSb. *Inorg. Chem.* **58**, 1826–1833 (2019).
31. W. Hu, S. Ye, Q. Li, B. Zhao, M. Hagihala, Z. Dong, Y. Zhang, J. Zhang, S. Torri, J. Ma, B. Ge, J. Luo, Strategic Design and Mechanistic Understanding of Vacancy-Filling Heusler Thermoelectric Semiconductors. *Adv. Sci.* **11**, 2407578 (2024).
32. K. S. Kim, Y. Kim, H. Mun, J. Kim, J. Park, A. Y. Borisevich, K. H. Lee, S. W. Kim, Direct Observation of Inherent Atomic-Scale Defect Disorders responsible for High-Performance $Ti_{1-x}Hf_xNiSn_{1-y}Sb_y$ Half-Heusler Thermoelectric Alloys. *Adv. Mater.* **29**, 1702091 (2017).
33. H. J. Lee, K. H. Lee, L. Fu, G. Han, H.-S. Kim, S.-I. Kim, Y.-M. Kim, S. W. Kim, Critical role of atomic-scale defect disorders for high-performance nanostructured half-Heusler thermoelectric alloys and their thermal stability. *Acta Mater.* **180**, 97–104 (2019).
34. H. Zhu, W. Li, A. Nozariasbmarz, N. Liu, Y. Zhang, S. Priya, B. Poudel, Half-Heusler alloys as emerging high power density thermoelectric cooling materials. *Nat. Commun.* **14**, 3300 (2023).
35. J. E. Saal, S. Kirklin, M. Aykol, B. Meredig, C. Wolverton, Materials Design and Discovery with High-Throughput Density Functional Theory: The Open Quantum Materials Database (OQMD). *JOM* **65**, 1501–1509 (2013).
36. S. Kirklin, J. E. Saal, B. Meredig, A. Thompson, J. W. Doak, M. Aykol, S. Rühl, C. Wolverton, The Open Quantum Materials Database (OQMD): assessing the accuracy of DFT formation energies. *npj Comput. Mater.* **1**, 15010 (2015).
